# Supplementary material for: A SARS-CoV-2 variant‑adjusted threshold of protection model for monoclonal antibody pre-exposure prophylaxis against COVID-19
Source: Nat Commun. 2025 Oct 14;16:9101. doi: 10.1038/s41467-025-63972-4 (PMC12521407; doi:10.1038/s41467-025-63972-4)
Supplement: Supplementary file 1 — Supplementary Information [file 41467_2025_63972_MOESM1_ESM.pdf]

Supplementary Information

A SARS-CoV-2 variant-adjusted threshold of protection model for monoclonal antibody pre-exposure prophylaxis against COVID-19

Rhiannon Edge*, PhD, Sam Matthews*, MSc, Bahar Ahani, BS, Anastasia A. Aksyuk, PhD, Lindsay Clegg, PhD, John L. Perez, MD, MBA, MA, Mark T. Esser, PhD, Lee-Jah Chang, MD, Ian Hirsch, PhD, Tonya Villafana, PhD, John Pura, PhD, Oleg Stepanov, MS, Katie Streicher, PhD, Tom White, PhD, Taylor S. Cohen, PhD, Dean Follmann, PhD, Peter B. Gilbert, PhD, Seth Seegobin, PhD

*These authors contributed equally.

This supplemental information has been provided by the authors to give readers additional information about the work.

Table of Contents

Table of Contents 2
Supplemental Methods 4
Assessment of serum tixagevimab and cilgavimab concentrations and in vitro 50% inhibitory concentration (IC_{50}) measurements as a predictor of neutralising antibody (nAb) 50% inhibitory dilution (ID_{50}) titres 4
Prevalence of variants over time within each study 5
Derivation of prevalence-adjusted predicted nAb ID_{50} titres 7
PROVENT threshold of protection (ToP) model 8
Assessment of ToP model parameterisation 9
Transformation of prevalence-adjusted nAb ID_{50} titres 11
External assessment of instantaneous efficacy from the PROVENT ToP model using SUPERNOVA 13
External assessment of average efficacy from the PROVENT ToP model using SUPERNOVA 14
PROVENT + SUPERNOVA ToP model 17
Application of the ToP model 18
Table S1: PROVENT + SUPERNOVA ToP model estimates 22
Table S2: Model selection table from PROVENT ToP models 23
Table S3: AIC under different transformations of prevalence-adjusted nAb ID_{50} titres 24
Fig. S1: Prevalence of SARS-CoV-2 variants in circulation during PROVENT (**A**) and prevalence-adjusted nAb ID_{50} titre over time with COVID-19 cases (**B**) 25
Fig. S2: Prevalence of SARS-CoV-2 variants in circulation during SUPERNOVA with ≥5% on-study prevalence (**A**) and prevalence-adjusted nAb ID_{50} titre over time with COVID-19 cases (**B**) 26
Fig. S3: The profile of prevalence-adjusted nAb ID_{50} titres informing the PROVENT + SUPERNOVA ToP model 27
Fig. S4: Concordance between observed and predicted nAb ID_{50} titres 28
Fig. S5: Restricting the PROVENT ToP model to non-negative efficacies 29
Fig. S6: Threshold curves considered during model selection from PROVENT ToP models 30
Fig. S7: External assessment of overall efficacy (instantaneous [**A**] and average [**B**]) from PROVENT ToP model with no intercept using SUPERNOVA data 31
Fig. S8: Concordance between observed SUPERNOVA efficacies and PROVENT ToP model–derived predictions for alternative model parameterisations (with no intercept [**A**] and with nAb ID_{50} titre intercept [**B**]) 32

Fig. S9: Internal assessment of overall efficacy from PROVENT ToP model based on dominant variant 34
Fig. S10: PROVENT ToP model derived variant specific efficacy of sipavibart 35
References 36

Supplemental Methods

Assessment of serum tixagevimab and cilgavimab concentrations and in vitro 50% inhibitory concentration (IC_{50}) measurements as a predictor of neutralising antibody (nAb) 50% inhibitory dilution (ID_{50}) titres

Serum monoclonal antibody (mAb) concentrations of tixagevimab and cilgavimab, and nAb ID_{50} titres against different severe acute respiratory syndrome coronavirus 2 (SARS-CoV-2) variants of concern have been shown to correlate[1].

A serology study was conducted in a subset of matched samples from 300 PROVENT participants randomised to receive tixagevimab–cilgavimab, in which serum samples for SARS-CoV-2 nAb titres were taken on Days 29, 58 and 181 post dose of tixagevimab–cilgavimab. A subset of samples based on aliquot availability were selected for the post hoc analysis described in this publication.

Serum mAb concentrations of tixagevimab and cilgavimab were measured in these samples using a validated assay, with the tixagevimab–cilgavimab concentration calculated as the sum of concentrations of the individual mAbs. nAb titres were measured for Wuhan, Alpha, Delta, BA.1, BA.1.1, BA.2, and BA.4/5 at Monogram Biosciences in validated assays.

Using the observed serum mAb concentration, denoted as C, and the mAb in vitro IC_{50} values presented in Table 1, predicted nAb ID_{50} titres, $\hat{Y}$, were derived for each SARS-Cov-2 variant k, participant i at study day t_d (defined as number of days since first dose) from the following formula:

$$\hat{Y}_{i,t_d,k} = \frac{C_{i,t_d}}{\mathrm{IC}_{50_k}} \qquad \text{E 1}$$

Concordance between observed and predicted nAb ID_{50} titres was assessed in this subsample, censoring observations in accordance with the time-to-event analysis for symptomatic reverse transcription-polymerase chain reaction (RT-PCR)–confirmed coronavirus disease 2019 (COVID-19) (i.e., prior to SARS-CoV-2 vaccinations and infections). The purpose of this was to assess the validity of the vitro measurements of IC_{50} in a subset of our data. Lin's concordance correlation coefficient (CCC) and the respective 95% confidence intervals (CIs) were reported[2]. Paired samples of observed nAb ID_{50} titres and serum mAb concentrations (ng/mL) were considered when both were available and above the lower limit of quantification (LLoQ). As no quantifiable BA1.1 nAb ID_{50} titres were observed, BA1.1 was not included in this analysis.

Fig. S4 displays the plot of the observed versus predicted nAb ID_{50} titres. Concordance was low for BA.1 where there were limited observed values above the LLoQ. Otherwise, concordance ranged from 0.6 to 0.7.

Prevalence of variants over time within each study

The PROVENT study was conducted from November 21, 2020, to August 16, 2022. Contemporaneous surveillance data from the Global Initiative on Sharing All Influenza Data (GISAID) was selected based on study countries and World Health Organization (WHO) variants where both in vitro IC_{50} measurements were available, and variants were deemed to have ≥1% prevalence at any point during study follow-up[1]. The 16 selected WHO variants are presented in Table 2. Prevalence was calculated based

on Pango lineage and sublineage counts per WHO group from the selected variants to ensure unit summation at each calendar time and country. The resulting prevalence over study follow-up (defined as on-study prevalence) is displayed in Fig. S1A for time since dose.

Fig. S1A shows mean prevalence over study days. Dominant variants during study follow-up included Alpha, Delta and Omicron BA.1.1. In vitro data show that tixagevimab–cilgavimab had strong potency against Alpha and Delta, but reduced potency against Omicron and its sublineages (particularly BA.1 and BA.1.1). As any analysis that relies on nAb titres against a single variant could be misleading (Fig. S9), we derived a prevalence-adjusted nAb ID_{50} titre.

At the time of analysis, the SUPERNOVA study was ongoing. SUPERNOVA started in April 2023 and the reporting period for the analysis presented here ended in March 2024. Similarly to PROVENT, GISAID data was used to evaluate the prevalences of SARS-COV-2 variants in circulation during the study period. However, due to the evolution of the virus, there were more variants and therefore the prevalence selection criteria were slightly modified. Variants with ≥5% prevalence at any point during study follow-up were selected. Additionally, IC_{50} values were measured using viruses with distinct spike sequence, therefore Hedgehog lineages were used to group Pango lineages sharing the same spike sequence and prevalence was calculated per Hedgehog lineage. For some variants, the IC_{50} value was assumed based on the spike protein mutations affecting the sipavibart binding site (L455X and F456X) and impacting potency, in the absence of an available in vitro measurement. Variants with F456X and L455X mutations are imputed to a value of 1000 ng/mL

(equal to the upper assay limit) and 83.1 ng/mL (consistent with JN.1), respectively, otherwise imputed to 7.1 ng/mL, which represents an average of known in vitro IC_{50} values for sipavibart against variants that do not have either F456X or L455X mutations. Viral prevalence was calculated based on lineage counts from the selected variants to ensure unit summation at each calendar time and continent. Lineages were converted from Hedgehog to Pango (Table 2). On-study prevalence is displayed in Fig. S2A or time since dose for those variants that obtain a ≥5% on-study prevalence.

Derivation of prevalence-adjusted predicted nAb ID_{50} titres

Region was defined on a country level for PROVENT and continent level for SUPERNOVA. The $\log_{10}$-transformed prevalence-adjusted IC_{50} (ng/mL), denoted as $\widetilde{\mathrm{IC}}_{50_{t_c,j}}$, was derived using prevalence weights w for each region j and variant k at calendar time t_c as follows:

$$\log_{10}\left(\widetilde{\mathrm{IC}}_{50_{t_c,j}}\right) = \frac{\sum_k w_{t_c,j,k} \log_{10} \mathrm{IC}_{50_k}}{\sum_k w_{t_c,j,k}} \quad \text{E 2}$$

Once the prevalence-adjusted IC_{50} was back transformed this was equivalent to the formulation for the weighted geometric mean. The prevalence-adjusted IC_{50} was combined by calendar date and region with individual daily serum mAb concentration (ng/mL) predictions from a population pharmacokinetic (popPK) model, denoted $\tilde{c}_{i,t_c}$. Similar to (E 1) prevalence-adjusted nAb ID_{50} titres, $\tilde{Y}_{i,t_c}$, were derived as:

$$\tilde{Y}_{i,t_c} = \frac{\tilde{c}_{i,t_c}}{\widetilde{\mathrm{IC}}_{50_{t_c,j}}} \quad \text{E 3}$$

PROVENT threshold of protection (ToP) model

As nAb ID_{50} titre levels change over time, they become a time-varying covariate in modelling the association between predicted nAb ID_{50} titre and risk of symptomatic COVID-19. Of the available options for modelling time-dependent covariate data, the time-varying Cox model incorporates time-varying covariates while allowing for censored observations.

Using the data from PROVENT, we fitted a Cox model with a time-varying covariate to explore the risk of symptomatic RT-PCR–confirmed COVID-19 cases through day 366, as a function of day-level prevalence-adjusted nAb ID_{50} titre, $\tilde{Y}_{i,t_d}$ (calendar date mapped to study day). Although the risk of event for the placebo group was captured in the baseline hazards, we imputed placebo titre as zero for all time points. However, nAb ID_{50} titres are added to the model by their interaction with treatment, which is zero for placebo, therefore the placebo titres have no effect on the model fit.

The time-varying Cox model assumes the hazard for participant i as:

$$\mathrm{h}\big(t_d; Z_i, \mathrm{X_i}(t_d)\big) = \mathrm{h}_0(t_d) exp(Z_i[\gamma_0 + \gamma_1 \mathrm{X_i}(t_d)]) \text{ ,} \qquad \text{M 1}$$

where: $\mathrm{h}_0(t)$ represents the baseline hazard function, Z_i is the treatment indicator (equals one for individuals randomised to tixagevimab–cilgavimab and zero otherwise) and $\mathrm{X}_i(t_d) = \log_{10}\big(\tilde{Y}_{i,t_d} + 1\big)$ is a time-varying covariate (transformation assessed below).

The model estimates γ_0 and γ_1 are presented in Table S2. The corresponding covariance matrix for (M 1) is:

$$\boldsymbol{\Sigma}_{\boldsymbol{\gamma}} = \begin{bmatrix} \sigma_{\gamma_0}^2 & \rho\sigma_{\gamma_0}\sigma_{\gamma_1} \\ \rho\sigma_{\gamma_0}\sigma_{\gamma_1} & \sigma_{\gamma_1}^2 \end{bmatrix} = \begin{bmatrix} 0.127 & -0.036 \\ -0.036 & 0.014 \end{bmatrix}$$

Assessment of ToP model parameterisation

To additionally check the model fit under the assumption of zero efficacy at zero titre, the ToP model was further simplified to:

$$\mathrm{h}\big(t_d; Z_i, \mathrm{X}_i(t_d)\big) = \mathrm{h}_0(t_d)\exp\big(\gamma_1 Z_i \mathrm{X}_i(t_d)\big) \qquad \text{M 2}$$

Parameter estimates from the ToP model with (M 1) and without an efficacy intercept (M 2) are presented in Table S2.

A likelihood ratio test comparing the ToP model with and without an efficacy intercept term was performed. The inclusion of γ_0 for improvement in the model fit is associated with the *P* value of 0.0743. This suggests some model fit is to be gained through the inclusion of γ_0 by not restricting the curve through the origin. Following similar methods used for Fig. 4 and Fig. 5 (described in a later section), the goodness of fit for the no intercept model was assessed against the instantaneous and average overall efficacy observed in SUPERNOVA. Fig. S7 shows this model over-estimates efficacy (i.e., is anti-conservative), showing a worst fit than the two-parameter model when compared to Fig. 4 and Fig. 5. The absolute mean difference between PROVENT ToP model predicted and observed efficacy is 12.6% following the one-parameter approach compared to 5.0% following the two-parameter approach when evaluating the overall average efficacy, and 13.4% following the one-parameter approach compared to 8.7% following the two-parameter approach when evaluating the overall instantaneous efficacy.

Inclusion of baseline covariates was explored based on their statistical significance (Table S2) and the resulting model was fitted:

$$\mathrm{h}(t_d; Z_i, \mathrm{X}_i(t_d), \mathrm{region}_i, \mathrm{sex}_i, \mathrm{BMI}_i) = \mathrm{h}_0(t_d)\exp(Z_i[\gamma_0 + \gamma_1 \mathrm{X}_i(t_d)] + \beta_1 \mathrm{region}_i + \beta_2 \mathrm{sex}_i + \beta_3 \mathrm{BMI}_i) \,, \qquad \text{M 3}$$

However, despite lowering the Akaike Information Criterion (AIC) by ≈ 43.6, this did not lead to substantial changes to the parameterisation of γ_0 or γ_1, resulting in a similar threshold curve (Fig. S6), thus, the more parsimonious model was chosen to allow for more generalisable applications.

The efficacy intercept in (M 1) allows the model to be flexible in not overfitting to the zero efficacy when nAb titres are not present. This results in negative efficacies relative to placebo which is not biologically plausible. One method (proposed during peer review) to restrict estimated efficacies to non-negative values whilst not confining the curve to the origin is to fit a model with an intercept on the nAb ID_{50} titre axis. This was done by fitting the following model:

$$\mathrm{h}\big(t_d; Z_i, \mathrm{X}_i(t_d)\big) = \mathrm{h}_0(t_d)\exp\big(\gamma_1 Z_i \mathrm{X}_i(t_d)\big) \qquad \text{M 4}$$

where:

$$\mathrm{X_i}(t_d) = \begin{cases} \log_{10}\big(\tilde{Y}_{i,t_d} - \delta + 1\big) & \text{if } \tilde{Y}_{i,t_d} > \delta \\ 0 \text{ otherwise} \end{cases}$$

and δ gives the nAb-titre intercept to be optimised. 5-fold cross validation was used to optimise δ for a range of candidate values between 0 to 150. When $\delta = 0$ this returns (M 2). Partition into each fold was stratified by treatment and events to retain the 2:1 randomisation ratio and the within-treatment distribution of events. Within each fold, the model fit on the current training set was used to estimate the average overall

efficacy at 365 days post dose in the remaining 20% of participants (the test set) for each candidate value of δ. The average δ that minimised the absolute mean difference in the model predicted and observed average efficacy from each fold was selected. The model was then refit on all PROVENT participants. The results of this optimisation algorithm and resulting threshold curve are displayed in Fig. S5.

One limitation of (M 4) is the threshold slope is optimised based on prevalence-adjusted nAb ID_{50} titres > 78.8. This leads to a reduction in event counts (118 to 84), resulting in wider confidence intervals.

Following the same approach as Fig. 7 (described in a later section), (M 4) was used to predict the variant-specific efficacies in SUPERNOVA. The concordance of these estimates vs the observed efficacies in SUPERNOVA are shown in Fig. S8, along with the comparison for the no intercept model described in (M 2). (M 4) has comparable concordance to (M 2) for 3 months post any dose, but worse concordance for 6 months post any dose. Both models result in worse concordance compared to (M 1) (Fig. 7).

Transformation of prevalence-adjusted nAb ID_{50} titres

The transformation $\log_{10}(\tilde{Y}_{i,t_d} + 1)$ was applied to preserve positivity at small nAb ID_{50} titres and normalise the distribution for participants in the active arms. For participants in the control arm the model is unaffected by the choice of the imputed value, as described (see section on PROVENT threshold of protection (ToP) model, above).

A problem with the transformation $\log_{10}(\tilde{Y}_{i,t_d}+1)$ is results are unstable under different parametrisations of $\tilde{Y}_{i,t_d}$. Consider a reparameterisation $Z_{i,t_d}=c\tilde{Y}_{i,t_d}$ for some constant c, such that

$$\tilde{Y}_{i,t_d}=\frac{10^{\left(\frac{(\log(\mathrm{HR})-\gamma_0)}{\gamma_1}\right)}-1}{c}. \quad \text{E 4}$$

As $\log_{10}(c\tilde{Y}_{i,t_d}+1)$ cannot be split out, this means the estimation of the HR depends on the choices of c. To overcome the stability issue, consider the following parameterisation of :

$$\mathrm{h}(t;Z_i,X_i)=\mathrm{h}_0(t)\exp\{Z_i[\gamma_0+\gamma_1\log_{10}(c\tilde{Y}_{i,t_d})]\} \quad \text{M 5}$$

with

$$\log\mathrm{HR}_i=\gamma_0+\gamma_1\log_{10}(c\tilde{Y}_{i,t_d})=[\gamma_0+\gamma_1\log_{10}(c)]+\gamma_1\log_{10}\tilde{Y}_{i,t_d}$$

Through the interaction with treatment, Z, and the addition of γ_0 stability is preserved under the new parametrisation $\beta_0=\gamma_0+\gamma_1\log_{10}(c)$ and $\beta_1=\gamma_1$. Therefore, the constant c does not need to be considered in the model expression. However, as $\log_{10}(\tilde{Y}_{i,t_d})$ is not restricted to the positive space (as $\log_{10}(\tilde{Y}_{i,t_d})<0$ when $\tilde{Y}_{i,t_d}<1$), and biological assumptions restrict $\gamma_1<0$ (decrease in efficacy is expected to follow a decrease in nAb ID_{50} titres), then (M 2) forces the efficacy curve through the point $(0,-\infty)$: leading to a loss in model flexibility.

Table S3 evaluates the AIC following the transformations $\log_{10}(\tilde{Y}_{i,t_d}+1)$ and $\log_{10}(\tilde{Y}_{i,t_d})$ being made to the prevalence-adjusted nAb ID_{50} titres based on fitting (M 1) to the PROVENT data. The $\log_{10}(\tilde{Y}_{i,t_d}+1)$ was chosen as this lowered the AIC by ≈ 4.7.

External assessment of instantaneous efficacy from the PROVENT ToP model using SUPERNOVA

Prevalence-adjusted nAb ID_{50} titres were derived from SUPERNOVA using daily serum sipavibart concentration predictions from a popPK model,[2] variant surveillance data, and IC_{50}s from (E 2) and (E 3).

The set $T_d = t_{d_{min}}, t_{d_{min}} + 1, \ldots, t_{d_{max}}$ is defined, where $t_{d_{min}}$ and $t_{d_{max}}$ denote the earliest and latest event times from SUPERNOVA in days since first dose. For each study day $t_d \in T_d$, the geometric mean of the prevalence-adjusted nAb ID_{50} titres at each timepoint, denoted as $\hat{Y}_{t_d}$, is calculated as

$$\hat{Y}_{t_d} = \exp\left(\frac{\sum_{i=1}^{n_{t_d}} \log\left(\tilde{Y}_{i,t_d}\right)}{n_{t_d}}\right) \tag{E 5}$$

where n_{t_d} is the number of participants at-risk on each study day.

The PROVENT ToP model is used to evaluate the efficacy (E) for each timepoint from:

$$\mathrm{E}_1(t_d) = 100\left(1 - \exp\left(\gamma_0 + \gamma_1 \mathrm{X}_i(t_d)\right)\right) \tag{E 6}$$

and two-sided 95% confidence limits from:

$$100\left(1 - \exp\left(\gamma_0 + \gamma_1 \mathrm{X}_i(t_d) \pm Z_{0.975} \times \sqrt{\boldsymbol{\gamma}\boldsymbol{\Sigma}_{\boldsymbol{\gamma}}\boldsymbol{\gamma}^{\mathbf{T}}}\right)\right) \tag{E 7}$$

where: $\boldsymbol{\gamma} = [\gamma_0 \quad \gamma_1]$ and $\mathrm{X}_i(t_d) = \log_{10}\left(\hat{Y}_{t_d} + 1\right)$.

The daily Epanechnikov kernel-smoothed hazard functions are calculated for each arm. The bandwidth is optimised in each arm such that it minimises the mean

integrated squared error from the time of first dose up to the latest event time in that arm[3]. The observed daily efficacy is estimated as 100(1 – hazard ratio) (%).

Fig. 4 compares (E 6) and (E 7) to the estimate of the observed daily efficacy.

External assessment of average efficacy from the PROVENT ToP model using SUPERNOVA

The geometric mean of the prevalence-adjusted nAb ID_{50} titre up to each study day t_d was calculated evaluating:

$$\bar{Y}_{t_d} = \exp\left(\frac{\sum_{j=1}^{d}\sum_{i=1}^{n_{t_d}} \log\left(\tilde{Y}_{i,t_j}\right)}{\sum_{j=1}^{d} n_{t_j}}\right) \tag{E 8}$$

The PROVENT ToP model was used to evaluate the average efficacy from (E 6) and two-sided 95% confidence limits from (E 7) where instead $\mathrm{X}_i(t_d) = \log_{10}\left(\bar{Y}_{t_d} + 1\right)$.

Let τ denote the event/censoring time and δ = 1 for participants with an event, or otherwise 0. For each study day t_d the data was iteratively censored for $\tau_i > t_d$ by setting $\tau_i = t_d$ and $\delta = 0$. The relative risk and two-sided 95% CI was estimated from a Poisson regression with robust variance[4], including an offset for $\log(\tau_i)$ and adjusting for actual study intervention (sipavibart or comparator) and randomisation stratification factors. Randomisation stratification factors were COVID-19 vaccination status within 6 months before randomisation (Yes or No), SARS-CoV-2 infection within 6 months before randomisation (Yes or No), and tixagevimab–cilgavimab use within 12 months before randomisation (Yes or No). Observed efficacy was defined as 100(1 – relative risk) (%) of sipavibart versus comparator. This approach is

consistent with that prespecified for the efficacy analysis in the main cohort of the SUPERNOVA parent study[2].

For each study day, Fig. 5 compares the estimate of the average efficacy to the observed efficacy.

To further validate the PROVENT ToP model, the SUPERNOVA study results for efficacy attributable to overall variant, F456L/non-F456L variant, and non-F456L–specific subvariant analyses for 90 and 180 days post any dose[2] were compared with the PROVENT ToP model estimates for the same endpoints in Fig. 6. When the efficacy estimate was attributable to a single variant based on the set of subvariants circulating during follow-up then the model estimates were derived by first dividing the daily serum mAb concentration estimates in the at-risk set by the relevant monogram IC_{50} for the single prevalent variant to obtain the predicted nAbs. This was the case for the JN.1 + and BA.2.86 + subvariant analyses which were dominated by JN.1 (IC_{50} = 83.1 ng/mL) and BA.2.86 (IC_{50} = 3.8 ng/mL), respectively. A similar approach was taken for efficacy attributable to variants with a F456L mutation as those were considered to have an IC_{50} of 1000 (similar to the approach taken for variants with a F456X mutation). When the efficacy endpoint was attributable to a mixture of circulating variants (as in all other cases), the GISAID prevalence data for these sets of variants was separately merged onto at-risk study data by region and calendar time, and prevalence-adjusted nAb ID_{50} titres were derived following (E 2) and (E 3). Once this set of predicted, or prevalence-adjusted nAb ID_{50} titres were mapped to the at-risk population over the applicable follow-up period, the geometric average of

these was calculated using (E 8) and (E 6) and (E 7) were evaluated to calculate the PROVENT ToP model-based efficacy estimates and two-sided 95% CIs.

The point estimates of the PROVENT ToP model predictions of the SUPERNOVA endpoints and those reported for the SUPERNOVA study were assessed for agreement in Fig. 7 both visually (compared to the line $y = x$) and using Lin's CCC separately for the 3 and 6 months post any dose timepoints. Under the standard approach a Fisher's Z transformation is used for the confidence interval;[2] however, this assumes that the data follows an approximate normal distribution. As this assumption is not met, a bootstrap approach was taken. The steps are detailed below:

1. For $m = 1, \ldots, 1000$ participants were resampled with replacement with the number of participants receiving sipavibart (N=1631) and comparator (N=1649) fixed.
2. For each resample the following steps were repeated:
   - 2.1 Using the set of predicted, or prevalence-adjusted nAb ID_{50} titres in the at-risk set of participants for each endpoint (overall, matched non-F456L–containing variants, subvariant analyses, F456L-containing variants) and timepoint (3 or 6 months post any dose) defined above in this section, a resample was drawn based on those participants defined in 1.

   For each endpoint $e = 1, \ldots, 6$ and timepoint $t = 1, 2$:
     - 2.1.1 The geometric mean of the predicted, or prevalence-adjusted nAb ID_{50} titres was calculated using (E 8).

2.1.2 The PROVENT ToP model was evaluated based on the value from 2.1.1 using (E 6) to estimate the predicted efficacy, $p_{t,e,m}$.

2.2 Using the participant-level at-risk data a resample was drawn based on those participants in 1, where each resample with replacement was considered to be a separate participant.

For each endpoint $e = 1, \dots, 6$ and timepoint $t = 1, 2$:

2.2.1 The relative risk was estimated from a Poisson regression with robust variance,[4] including an offset of follow-up time and adjusting for planned study intervention (sipavibart or comparator) and randomisation stratification factors. Randomisation stratification factors were COVID-19 vaccination status within 6 months before randomisation (Yes or No), SARS-CoV-2 infection within 6 months before randomisation (Yes or No), and tixagevimab–cilgavimab use within 12 months before randomisation (Yes or No).

2.2.2 The observed efficacy, $o_{t,e,m}$ was estimated as 100(1 – relative risk).

2.3 Lin's CCC, $l_{t,m}$ between $p_{t,e,m}$ and $o_{t,e,m}$ was calculated separately for each timepoint.

2.4 The lower and upper two-sided 95% confidence limits for Lin's CCC were calculated as the 2.5th and 97.5th percentiles of $l_{t,m}$ separately for each timepoint.

PROVENT + SUPERNOVA ToP model

The ToP model was updated in (M 6) to include data from both PROVENT and SUPERNOVA:

$$\mathrm{h_s}\big(t_d; Z_i, \mathrm{X}_i(t_d)\big) = \mathrm{h_{0s}}(t_d)\exp(Z_i[\theta_0 + \theta_1 \mathrm{X}_i(t_d)]) \qquad \text{M 6}$$

where $\mathrm{h_{0s}}(t)$ represents the baseline hazard function for each study, Z_i is the treatment indicator (equals one for individuals randomised to tixagevimab–cilgavimab on PROVENT or sipavibart on SUPERNOVA, and zero otherwise), $\mathrm{X}_i(t_d) = \log_{10}\big(\tilde{Y}_{i,t_d} + 1\big)$ is a time-varying covariate, and $\tilde{Y}_{i,t_d}$ is the prevalence-adjusted nAb ID_{50} titre.

The model estimates γ_0 and γ_1 are presented in Table S1. The corresponding covariance matrix for (M 6) is:

$$\mathbf{\Sigma_\theta} = \begin{bmatrix} \sigma_{\theta_0}^2 & \rho\sigma_{\theta_0}\sigma_{\theta_1} \\ \rho\sigma_{\theta_0}\sigma_{\theta_1} & \sigma_{\theta_1}^2 \end{bmatrix} = \begin{bmatrix} 0.127 & -0.036 \\ -0.036 & 0.014 \end{bmatrix}$$

The efficacy-nAb titre relationships for models (M 1) and (M 6) are plotted in Fig. 8.

The ToP model was based on a time-varying Cox regression model. Under this model, the partial likelihood function was evaluated at each event time using all participants at risk. To fully understand the profile of prevalence-adjusted nAb ID_{50} titres informing the model, the prevalence-adjusted nAb ID_{50} titres in the at-risk set at all event times are presented in Fig. S3 for each study. It is seen that data from PROVENT informs the tails, whereas data from SUPERNOVA fills in the gap in PROVENT attributable to the Omicron crash in prevalence-adjusted nAb ID_{50} titres.

Application of the ToP model

The development of new mAbs targeting SARS-CoV-2 is crucial given the current and possible future variant landscape. Rapid clinical evaluation of new mAbs is necessary to bring effective mAbs to patients quickly, as the virus evolves and the

effectiveness of previously authorised mAbs decreases. mAb concentrations and in vitro IC_{50} values allow for rapid assessments of expected efficacy against novel SARS-CoV-2 variants. Additionally, these predicted nAb levels are related specific to the administered mAb as serum mAb concentration is not affected by increases in nAb levels due to vaccinations or infections. Using this framework, a non-inferiority assessment of nAb levels against a contemporary variant can be made to a desired efficacy level.

Consider the PROVENT ToP model defined in (M 1). For a target variant k, the minimal serum mAb concentration c_{min} to achieve the desired efficacy level E is given by:

$$c_{min} = \mathrm{IC}_{50_k}\left[10^{\left(\frac{(\log(1-E)-\gamma_0)}{\gamma_1}\right)} - 1\right] \quad \text{E 9}$$

Under regulatory guidance for pharmacokinetic bioequivalence studies[5], the two-sided 90% confidence limits must fall between 0.8 and 1.25 to reject the null hypothesis against a difference in compounds. This approach is expanded to a non-inferiority study for the minimal serum mAb concentration to obtain a required level of efficacy against a target variant. For the new mAb, the pharmacokinetics C is assumed to follow a log-normal distribution $\log_{10}(C) \sim N(\log_{10}(\mu_C), \sigma_C^2)$. Non-inferiority is declared when the two-sided 90% lower confidence limit comparing C to c_{min} is greater than 0.8. This is equivalent to:

$$\log_{10}(\mu_C) - \log_{10}(c_{min}) - Z_{0.95} \times \sqrt{\frac{\sigma_C^2}{n}} \geq \log_{10}(0.8) \quad \text{E 10}$$

with sample size formula:

$$n = \frac{Z_{0.95}^2 \times \sigma_C^2}{\left[\log_{10}\left(\frac{\mu_C}{c_{min} \times 0.8}\right)\right]^2} \qquad \text{E 11}$$

In (E 10), we implicitly make the assumption that μ_C and c_{min} are drawn from the same distribution, thus there is no contribution to the standard error associated with c_{min}. This is likely to be a conservative approach as c_{min} is derived from multiple studies and is therefore likely to have a relatively small standard error. The delta method could be applied to (E 9) to calculate variance under an approximate normal distribution (following from the central limit theorem) and weight the standard error as a function of this and the assumed variance for C. However, in practice, guideline values are generally not presented with their related uncertainty for clinical simplicity.

Define $\widehat{C_t}$ as the geometric mean of the serum mAb concentration through time t, following a similar approach to (E 8). Under the PROVENT + SUPERNOVA ToP model the average efficacy E over time t is minimally achieved against all variants with an IC_{50} up to $\mathrm{IC}_{50_{max}}$, where:

$$\mathrm{IC}_{50_{\max}} = \frac{\widehat{C_t}}{10^{\left(\frac{(\log(1-E)-\theta_0)}{\theta_1}\right)} - 1} \qquad \text{E 12}$$

A useful application of the ToP modelling approach is the assessment of predicted efficacy over time for existing mAbs against novel target SARS-Cov-2 variants. This can be used to infer duration of protection at a target efficacy level for currently circulating variants, and hence dynamically inform dosing strategies. For a mAb with an available serum mAb concentration and safety database, popPK models can be established, allowing for prediction of serum mAb concentrations over time for new dosing regimens or populations without conducting a new clinical trial. These serum

mAb concentrations can then be divided by the in vitro IC_{50} value for the SARS-CoV-2 variant of interest to estimate the nAb ID_{50} titre profile over time for this mAb. The average nAb ID_{50} titre profile up to or at a given timepoint can then be used to generate a point estimate (E 6) and two-sided 95% CI (E 7) for predicted average efficacy through that duration, or instantaneous predicted efficacy at that timepoint. This approach is demonstrated in Fig. S10 using the participant-level pharmacokinetic data in the at-risk set. By evaluating (E 12) for a specific mAb dose and viral variant, the predicted duration of protection for a target level of efficacy can be determined. Similarly, simulation-based analysis can also be performed to determine the target dose and redosing interval expected to be effective in the current variant landscape.

Table S1: PROVENT + SUPERNOVA ToP model estimates

Parameter	Estimates (SE)	***P*** **value**
θ_0	0.430 (0.225)	0.0563
θ_1	–0.409 (0.094)	<0.0001

The estimate is defined as the maximum likelihood estimate of the parameter. The standard error is defined as the square root of the corresponding diagonal element of the estimated covariance matrix. The P-value is based on the one-sided Chi-squared distribution with degrees of freedom equal to 1 where the test-statistic is the square of the estimate divided by its SE.

SE standard error.

Table S2: Model selection table from PROVENT ToP models

ToP Model	AIC	Parameter	Estimates (SE)	***P*** **value**
No intercept (M 1)	1764.193	γ_1	−0.314 (0.063)	<0.0001
Efficacy intercept (M 2)	1763.007	γ_0	0.622 (0.356)	0.0811
		γ_1	−0.494 (0.120)	<0.0001
Efficacy intercept + baseline covariates (M 3)	1720.567	γ_0	0.664 (0.366)	0.0695
		γ_1	−0.509 (0.124)	<0.0001
		Region*: Europe	0.750 (0.304)	0.0136
		Sex: Female	0.530 (0.192)	0.0057
		BMI (kg/m^2)	0.043 (0.009)	<0.0001

The estimate is defined as the maximum likelihood estimate of the parameter. The standard error is defined as the square root of the corresponding diagonal element of the estimated covariance matrix. The P-value is based on the one-sided Chi-squared distribution with degrees of freedom equal to 1 where the test-statistic is the square of the estimate divided by its SE.

*Region contains groups for Europe and Northern America.

AIC Akaike Information Criterion, BMI body mass index; SE standard error, ToP threshold of protection.

Table S3: AIC under different transformations of prevalence- adjusted nAb ID_{50} titres

Transformation	AIC
$\log_{10}\left(\tilde{Y}_{i,t_d}+1\right)$	1763.007
$\log_{10}\left(\tilde{Y}_{i,t_d}\right)$	1767.746

AIC Akaike Information Criterion, ID_{50} 50% inhibitory dilution, nAb neutralising antibody.

*Fig. S1: Prevalence of SARS-CoV-2 variants in circulation during PROVENT (**A**) and prevalence-adjusted nAb ID_{50} titre over time with COVID-19 cases (**B**)*

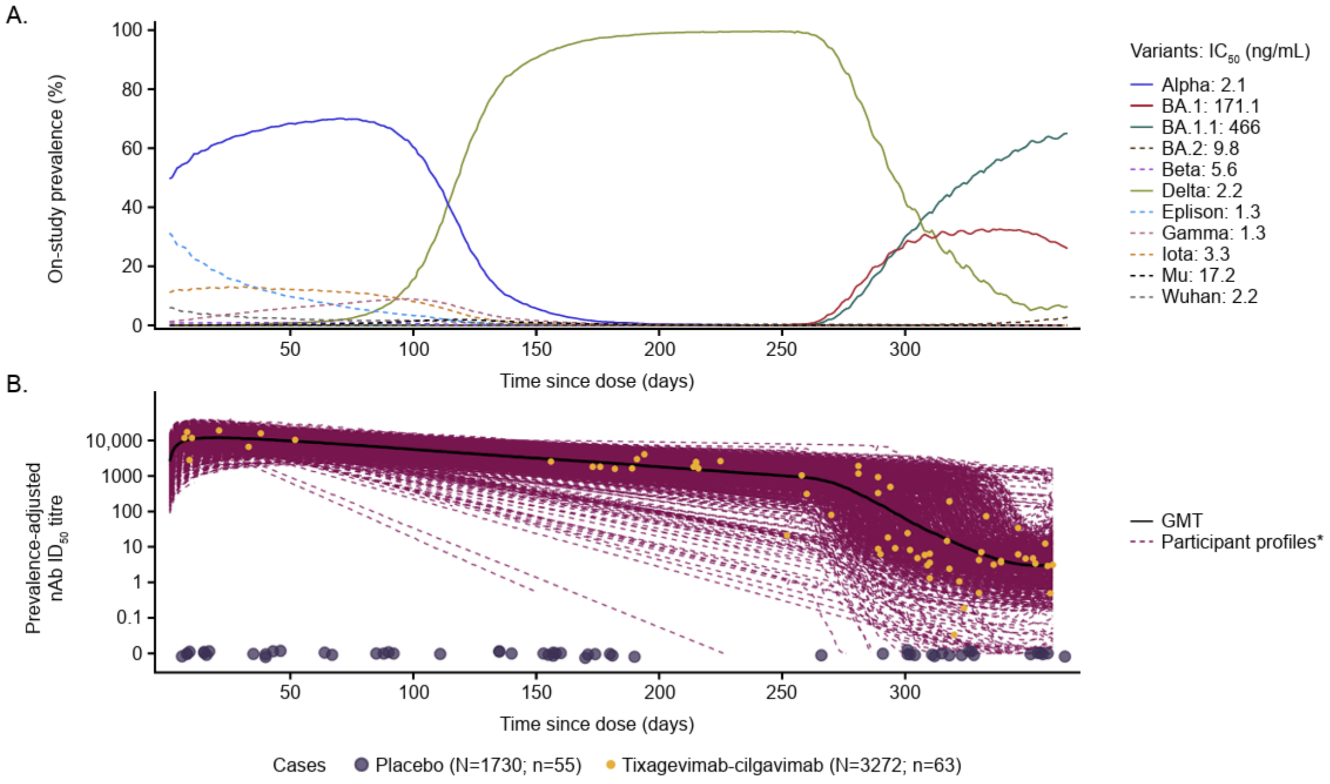

For display purposes, predicted nAb ID_{50} titres <0.01 are set as missing in the participant profiles.

*Dashed lines correspond to participant-level predicted prevalence-adjusted nAb ID_{50} titres at all days of follow-up and dots indicate values at event times (placebo dots [in purple] have had a slight jitter applied, are partially transparent to aid visualisation, and are plotted at twice the size of the tixagevimab–cilgavimab events to represent the 2:1 randomisation).

COVID-19 coronavirus disease 2019, GMT geometric mean titre, IC_{50} 50% inhibitory concentration, ID_{50} 50% inhibitory dilution, nAb neutralising antibody, N number of participants receiving each treatment, n number of participants with COVID-19 cases for each treatment, SARS-CoV-2 severe-acute-respiratory-syndrome-related coronavirus 2.

*Fig. S2: Prevalence of SARS-CoV-2 variants in circulation during SUPERNOVA with ≥5% on-study prevalence (**A**) and prevalence-adjusted nAb ID_{50} titre over time with COVID-19 cases (**B**)*

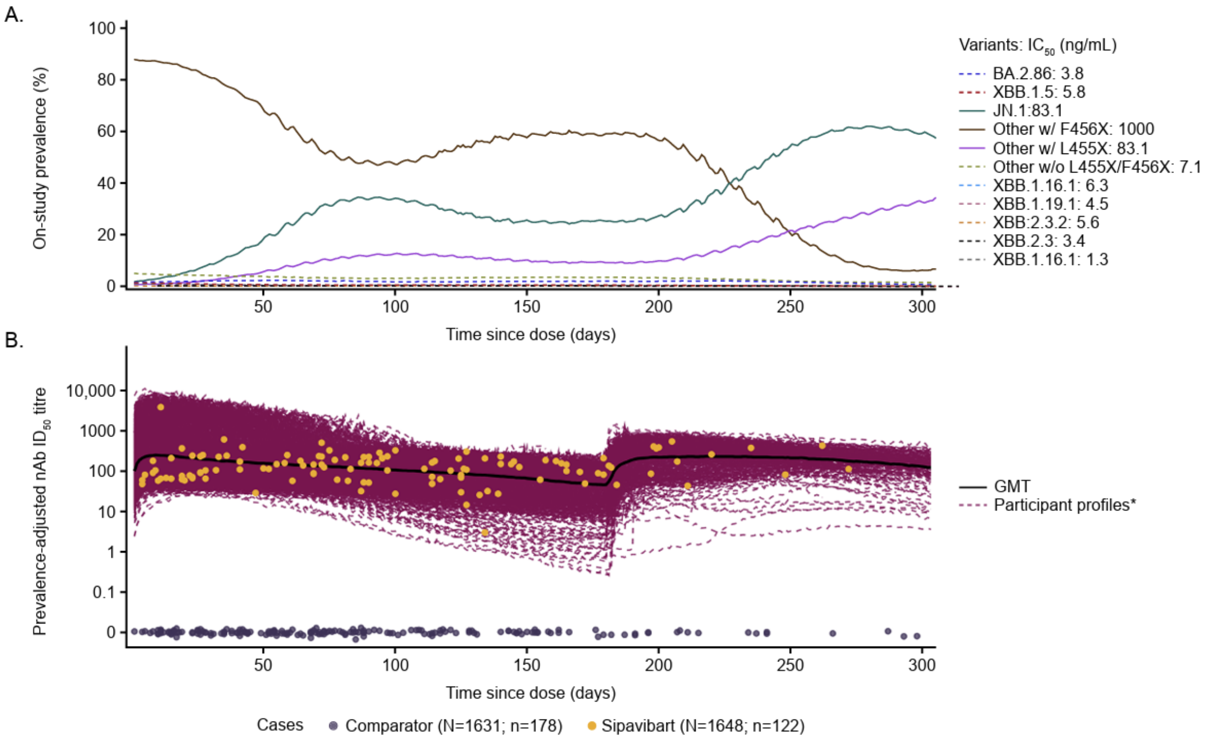

The comparator group includes participants who received tixagevimab–cilgavimab followed by placebo and those who received two doses of placebo.

*Dashed lines correspond to participant-level predicted prevalence-adjusted nAb ID_{50} titres at all days of follow-up and dots indicate values at event times (comparator dots [in purple] have had a slight jitter applied and are partially transparent to aid visualisation).

COVID-19 coronavirus disease 2019, GMT geometric mean titre, IC_{50} 50% inhibitory concentration, ID_{50} 50% inhibitory dilution, nAb neutralising antibody, N number of participants receiving each treatment, n number of participants with COVID-19 cases for each treatment, SARS-CoV-2 severe-acute-respiratory-syndrome-related coronavirus 2.

Fig. S3: The profile of prevalence-adjusted nAb ID_{50} titres informing the PROVENT + SUPERNOVA ToP model

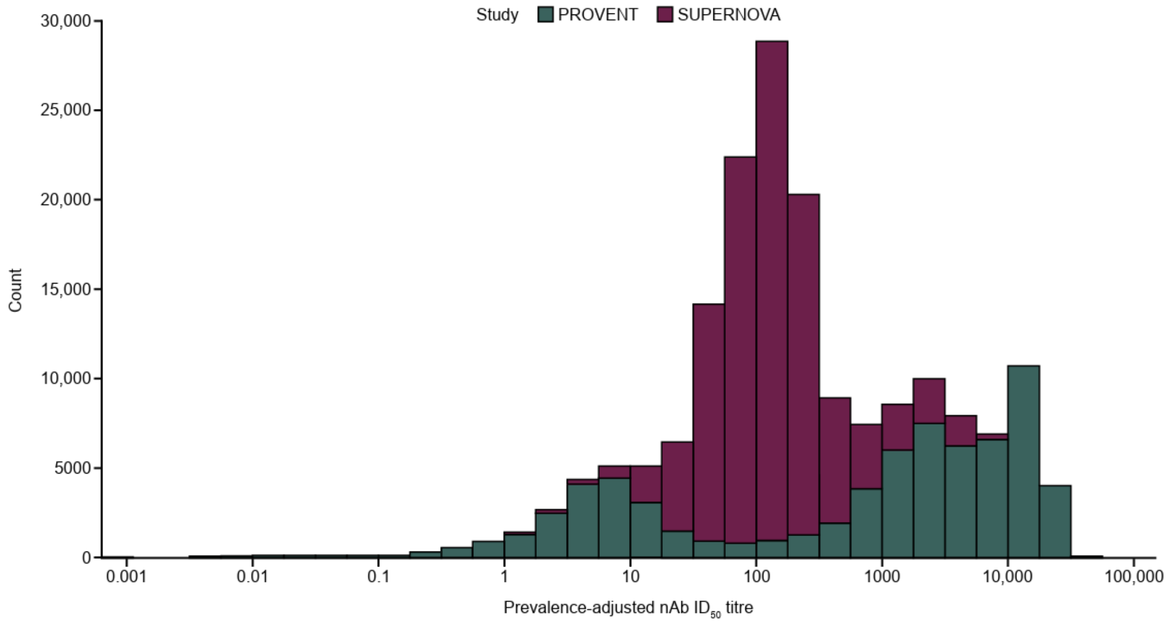

The profile of prevalence-adjusted nAb ID_{50} titres informing the PROVENT + SUPERNOVA ToP model. SUPERNOVA events strongly inform the region between 10–1000 but offer limited information for lower and higher titre level. By contrast, PROVENT data offer primary endpoint events across the full range of nAb ID_{50} titre levels observed within each study.

ID_{50} 50% inhibitory dilution, nAb neutralising antibody, ToP threshold of protection.

Fig. S4: Concordance between observed and predicted nAb ID_{50} titres

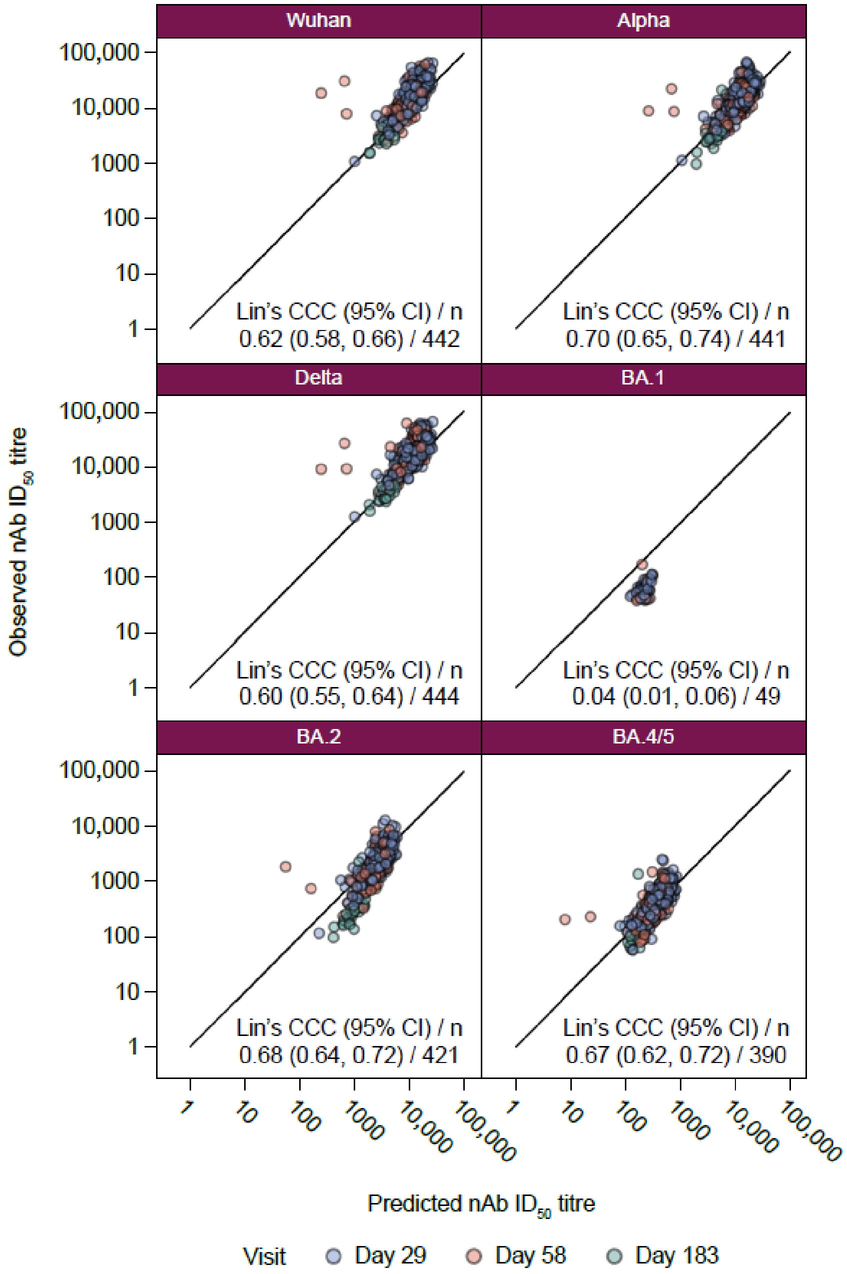

*The number of collected nAb ID_{50} titres above LLoQ over the follow-up time where serum mAb concentration is also available and above LLoQ.

Reference line of $y = x$ plotted to assess the agreement between observed and predicted nAb ID_{50} titres (log_{10} scale).

CCC concordance correlation coefficient, CI confidence interval, ID_{50} 50% inhibitory dilution, LLoQ lower limit of quantification, mAb monoclonal antibody, nAb neutralising antibody.

Fig. S5: Restricting the PROVENT ToP model to non-negative efficacies

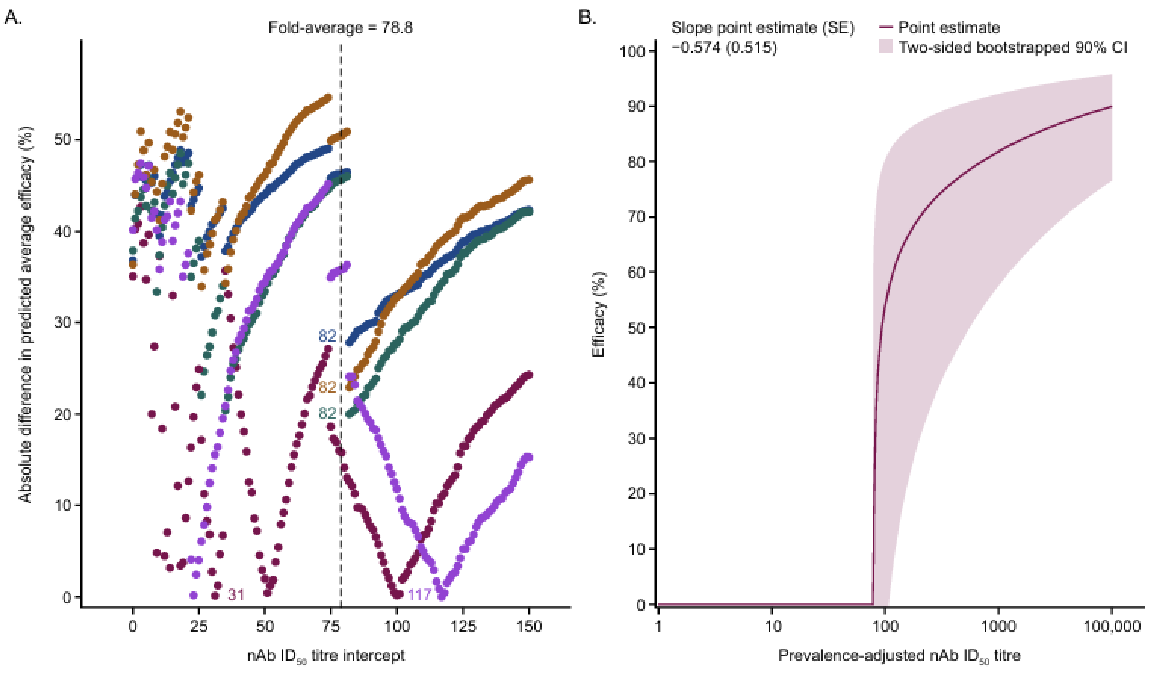

A time-varying Cox model was fit with an interaction between the treatment and $\log_{10}$(prevalence-adjusted nAb ID_{50} titres – nAb ID_{50} titre intercept +1) on the subset of the PROVENT data where prevalence-adjusted nAb ID_{50} titres > nAb ID_{50} titre intercept. When prevalence-adjusted nAb ID_{50} titres ≤ nAb ID_{50} titre intercept then efficacy is imputed to zero.

A: To optimise the nAb ID_{50} titre intercept the model 5-fold cross validation performed based on the participants in PROVENT for a range of candidate values. Within each fold: 1) the model fit to the training set was used to estimate the overall average efficacy at 365 days post dose in the remaining participants (the test set); 2) this value was compared to the overall average efficacy estimated as 100(1 – relative risk) (%), where the relative risk was evaluated with Poisson regression with robust variance, which includes treatment as a covariate and adjusted for the follow-up time at each day since first dose; 3) the nAb ID_{50} titre intercept value which minimized the absolute difference in these was selected.

B: Based on the fold-average of the nAb ID_{50} titre intercepts from A, the time-varying Cox model was refit on the full data. The slope point estimate is defined as the maximum likelihood estimate of the interaction term. The standard error is defined as the square root of the corresponding diagonal element of the estimated covariance matrix.

CI confidence interval, ID_{50} 50% inhibitory dilution, nAb neutralising antibody, SE standard error.

Fig. S6: Threshold curves considered during model selection from PROVENT ToP models

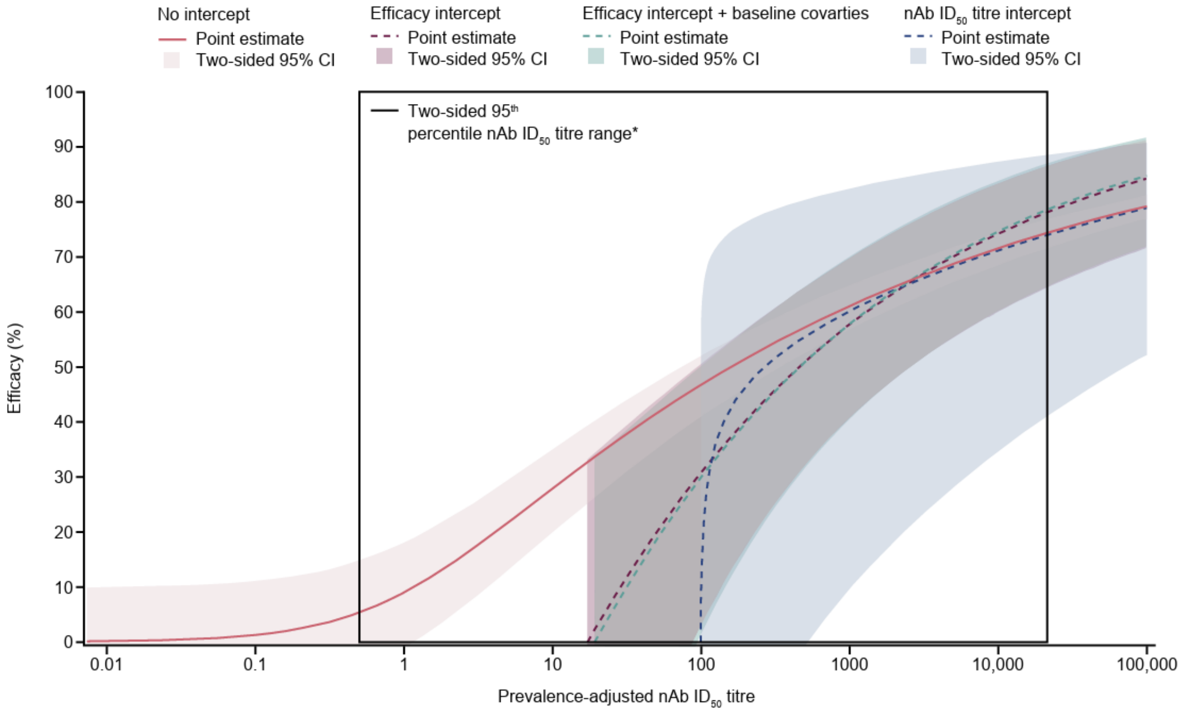

The time-varying Cox models defined in Table S2 and Fig. S5 were fit. Efficacy was calculated as 100(1 – HR) (%) based on the model parameter estimates. The corresponding the two-sided 90% CIs were calculated using the delta method. For the nAb ID_{50} titre intercept model these were imputed to zero when prevalence-adjusted nAb ID_{50} titre ≤ nAb ID_{50} titre intercept.

*The black box highlights the two-sided 95th percentile range of nAb ID_{50} titres informing the PROVENT ToP model.

HR hazard ratio, CI confidence interval, ID_{50} 50% inhibitory dilution, nAb neutralising antibody, ToP threshold of protection.

Fig. S7: External assessment of overall efficacy (instantaneous [A] and average [B]) from PROVENT ToP model with no intercept using SUPERNOVA data

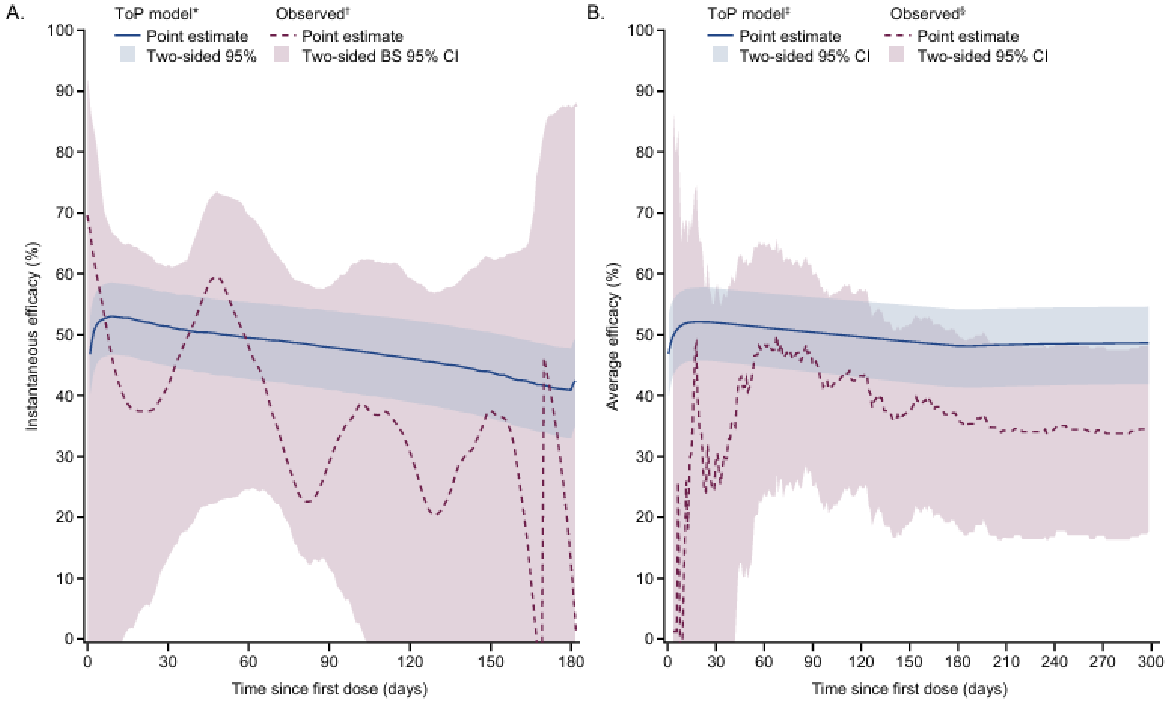

The ToP model was developed against PROVENT data using a time-varying Cox model with adjustment for the interaction between the treatment and $\log_{10}$(prevalence-adjusted nAb ID_{50} titres +1).

*The ToP model is used to estimate instantaneous overall efficacy and two-sided 95% CI through each day since first dose at the daily geometric average prevalence-adjusted nAb ID_{50} titre value at each timepoint after censoring participants for their second dose using SUPERNOVA study data.

†The daily Epanechnikov kernel-smoothed hazard functions were derived with an optimised window. From these the observed efficacy was estimated as 100(1 – HR) (%), with two-sided bootstrapped 95% CIs calculated from 1000 resamples with replacement.

‡The ToP model is used to estimate average overall efficacy and two-sided 95% CI through assessment at the average prevalence-adjusted nAb ID_{50} titre value up to each day since first dose using SUPERNOVA study data.

§The observed efficacy was defined based on actual intervention as 100(1 – relative risk) (%) of sipavibart versus comparator, where relative risk and the two-sided 95% CI were evaluated with a Poisson regression with robust variance, which includes treatment and randomisation stratification factors as covariates and adjusted for the follow-up time at each day since first dose. Randomisation stratification factors were COVID-19 vaccination status within 6 months before randomisation (Yes or No), SARS-CoV-2 infection within 6 months before randomisation (Yes or No), and tixagevimab–cilgavimab use within 12 months before randomisation (Yes or No).

BS bootstrapped, CI confidence interval, COVID-19 coronavirus disease 2019; HR hazard ratio, ID_{50} 50% inhibitory dilution, nAb neutralising antibody, ToP threshold of protection, SARS-CoV-2 severe-acute-respiratory-syndrome-related coronavirus 2.

*Fig. S8: Concordance between observed SUPERNOVA efficacies and PROVENT ToP model–derived predictions for alternative model parameterisations (with no intercept [**A**] and with nAb ID50 titre intercept [**B**])*

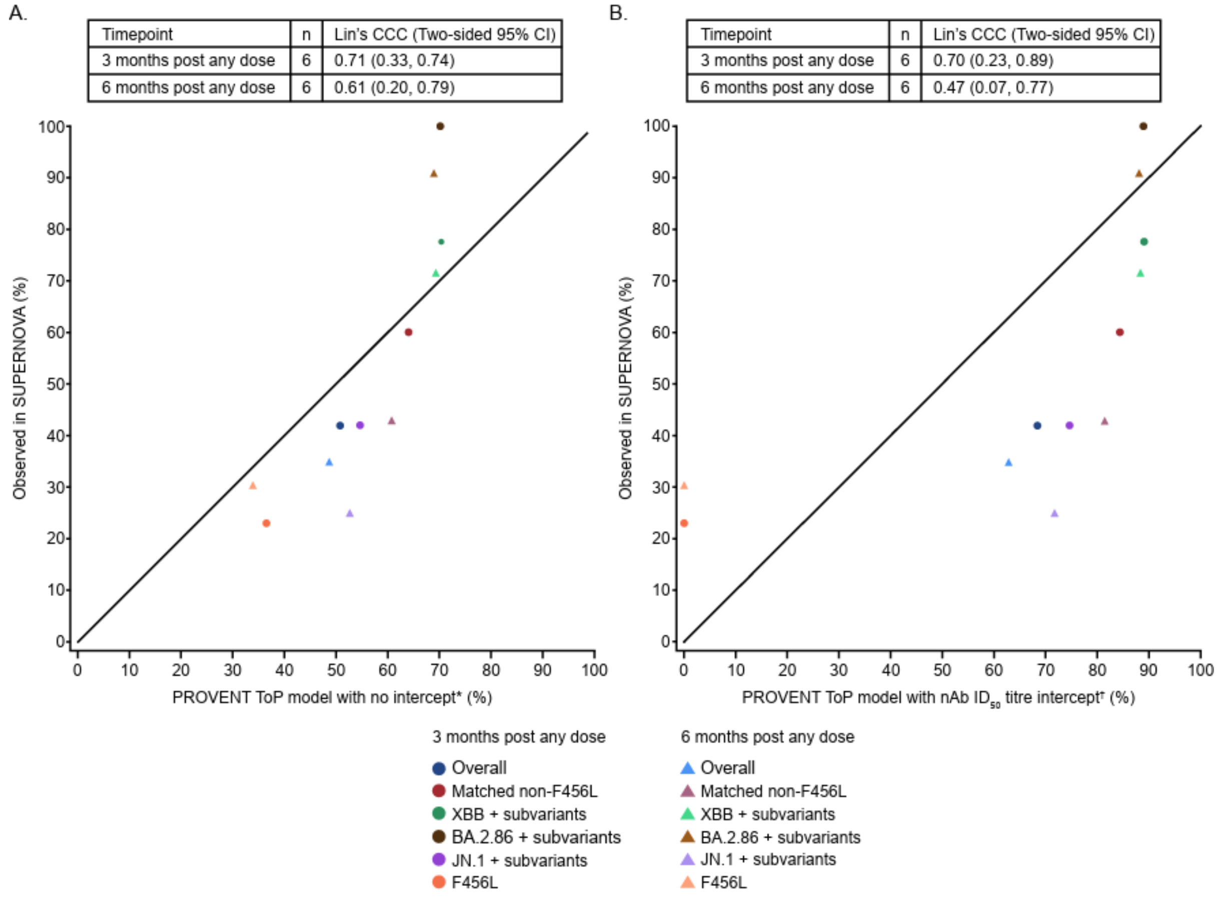

Two ToP models were developed against PROVENT data using a time-varying Cox model.

*The no intercept model adjusted for the interaction between the treatment and log_{10}(prevalence-adjusted nAb ID_{50} titres +1).

†The nAb ID_{50} titre intercept model adjusted for the interaction between the treatment and log_{10}(prevalence-adjusted nAb ID_{50} titres – nAb ID_{50} titre intercept +1) on the subset of the PROVENT data where prevalence-adjusted nAb ID_{50} titres > nAb ID_{50} titre intercept. When prevalence-adjusted nAb ID_{50} titres ≤ nAb ID_{50} titre intercept then efficacy is imputed to zero.

Observed SUPERNOVA efficacy was defined based on planned intervention as 100(1 – relative risk) (%) of sipavibart versus comparator, where relative were evaluated with Poisson regression with robust variance, which includes treatment and randomisation stratification factors as covariates and adjusted follow-up time. Randomisation stratification factors were COVID-19 vaccination status within 6 months before randomisation (Yes or No), SARS-CoV-2 infection within 6 months before randomisation, and tixagevimab–cilgavimab use within 12 months before randomisation.

The two ToP model estimates were used to predict the efficacy (%) based on actual intervention through evaluating the average nAb ID_{50} titres over the 90 days or 180 days post any dose according to the timepoint. For the overall endpoint, the average prevalence-adjusted nAb ID_{50} titres were considered after mapping GISAID prevalence data to at-risk participants for all variants with ≥5% prevalence. For the matched non-F456L and XBB+ subvariant endpoints a similar approach was followed with the exclusion of F456X variants (which are otherwise

assumed an IC_{50} of 1000), and the inclusion of only XBB subvariants based on hedgehog variants that included a XBB Pango lineage, respectively. Daily serum mAb concentrations estimated from population pharmacokinetics were used to estimate the predicted nAb ID_{50} titres in at-risk participants based on IC_{50} values of 3.8, 83.1, and 1000 ng/mL for BA.2.86, JN.1 subvariant analyses and variants with F456L mutations, respectively.

Reference line of $y = x$ plotted to visually assess the agreement.

CCC concordance correlation coefficient, CI confidence interval, IC_{50} 50% inhibitory concentration, ID_{50} 50% inhibitory dilution, ToP threshold of protection.

Fig. S9: Internal assessment of overall efficacy from PROVENT ToP model based on dominant variant

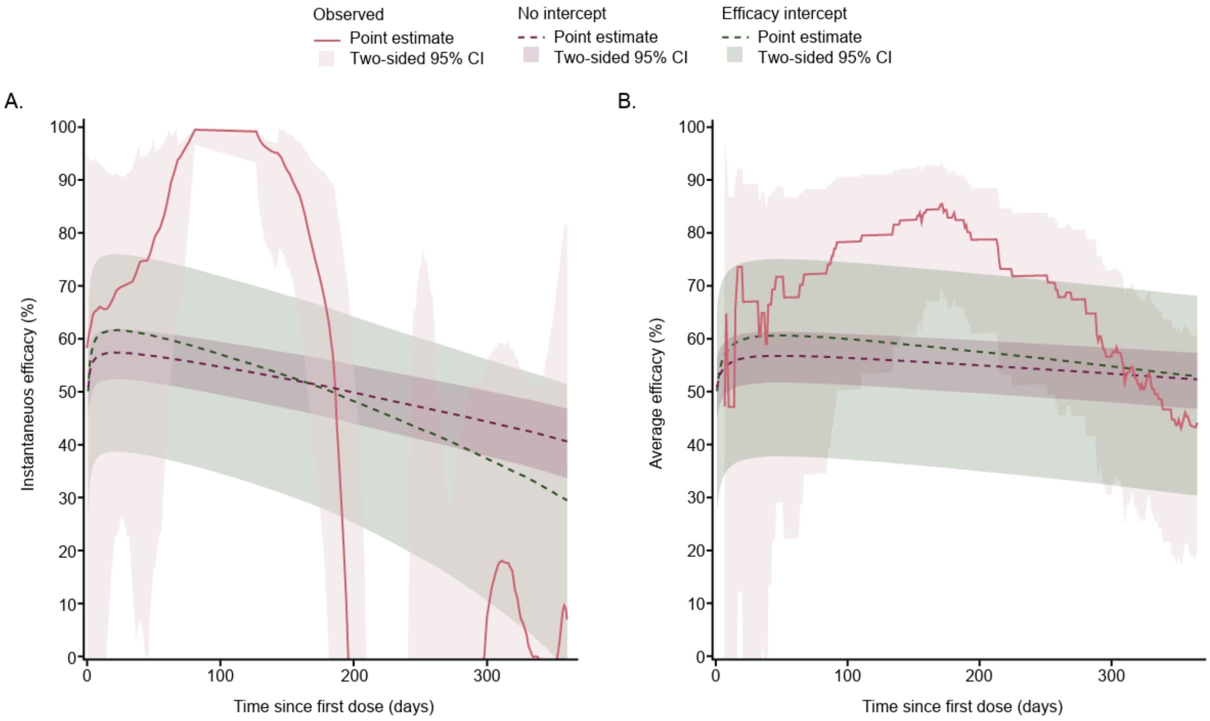

As observed in Fig. S1 SARS-CoV-2 variants Alpha and Delta are dominant over most of the study period (until approximately day 300), with similar in vitro IC_{50}s of 2.1 and 2.2 ng/mL, respectively. Given these similarities Delta predicted nAb ID_{50} titres are considered through the entire follow-up period.

Two ToP models were developed against PROVENT data using a time-varying Cox model. The no intercept model adjusted for the interaction between the treatment and $\log_{10}$(Delta predicted nAb ID_{50} titres +1). The efficacy intercept model additionally adjusted for the treatment.

A: The daily Epanechnikov kernel-smoothed hazard functions were derived with a 30-day window. From these the observed instantaneous overall efficacy (solid maroon line) was estimated as 100(1 – hazard ratio) (%), with two-sided bootstrapped 95% CIs (shaded maroon region) calculated from 1000 resamples with replacement. This was compared to the two ToP models, which were used to estimate instantaneous overall efficacy (no intercept model=short-dashed pink line; intercept model=medium-dashed green line) and two-sided 95% CI (no intercept model=shaded pink region; intercept model=shaded green region) through each day since first dose at the daily geometric average Delta predicted nAb ID_{50} titre value at each timepoint.

B: The observed average overall efficacy was defined based on actual intervention as 100(1 – relative risk) (%), where relative risk and the two-sided 95% CI were evaluated with a Poisson regression with robust variance, which includes treatment as a covariate and adjusted for the follow-up time at each day since first dose. This was compared to the two ToP models, which were used to estimate average overall efficacy (no intercept model=short-dashed pink line; intercept model=medium-dashed green line) and two-sided 95% CI (no intercept model=shaded pink region; intercept model=shaded green region) through each day since first dose at the average Delta predicted nAb ID_{50} titre value up to each day since first dose.

CI confidence interval, IC_{50} 50% inhibitory concentration, ID_{50} 50% inhibitory dilution, ToP threshold of protection.

Fig. S10: PROVENT ToP model derived variant s pecific efficacy of sipavibart

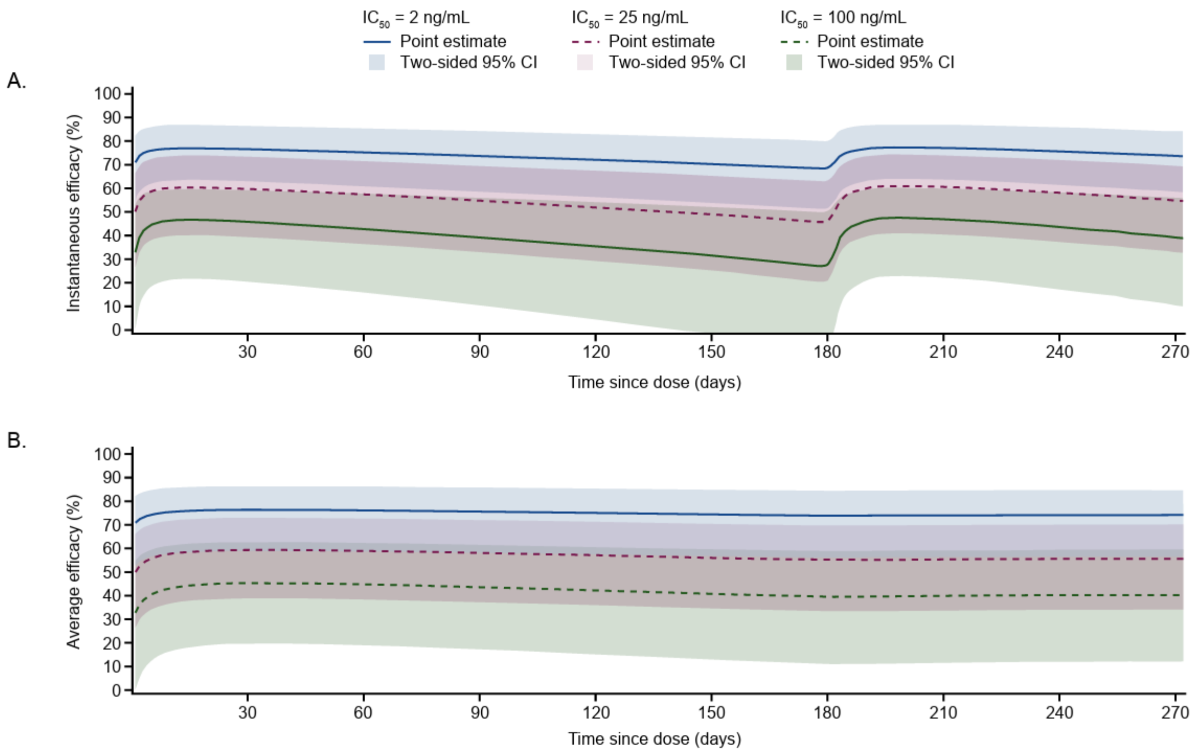

Participant-level predicted nAb ID_{50} titres were calculated by dividing the daily serum mAb concentration (ng/mL, estimated with the popPK model) by the in vitro IC_{50} values (ng/mL) presented in the legend using at-risk SUPERNOVA study data.

A: The PROVENT ToP model (derived from PROVENT study data) was used to estimate instantaneous efficacy (two-sided 95% CI) of sipavibart over time based on the average predicted nAb ID_{50} titre value at each time.

B: The PROVENT ToP model (derived from PROVENT study data) was used to estimate average efficacy (two-sided 95% CI) of sipavibart over time based on the average predicted nAb ID_{50} titre value up to each time.

CI confidence interval, IC_{50} 50% inhibitory concentration, ID_{50} 50% inhibitory dilution, mAb monoclonal antibody, nAb neutralising antibody, popPK population pharmacokinetic, ToP threshold of protection.

References

1 Shu, Y. & McCauley, J. GISAID: Global initiative on sharing all influenza data - from vision to reality. *Euro. Surveill.* **22**, 30494 (2017).

2 Haidar, G. et al. Efficacy and safety of sipavibart for prevention of COVID-19 in individuals who are immunocompromised (SUPERNOVA): a randomised, controlled, double-blind, phase 3 trial. *Lancet Infect. Dis.* **25**, 813-826 (2025).

3 Ramlau-Hansen, H. Smoothing Counting Process Intensities by Means of Kernel Functions. *Ann. Stat.* **11**, 453-466 (1983).

4 Zou, G. A modified poisson regression approach to prospective studies with binary data. *Am. J. Epidemiol.* **159**, 702-706 (2004).

5 Davit, B. M. et al. Implementation of a reference-scaled average bioequivalence approach for highly variable generic drug products by the US Food and Drug Administration. *AAPS J.* **14**, 915-924 (2012).
