# Supplementary material for: A SARS-CoV-2 variant‑adjusted threshold of protection model for monoclonal antibody pre-exposure prophylaxis against COVID-19
Source: Nat Commun. 2025 Oct 14;16:9101. doi: 10.1038/s41467-025-63972-4 (PMC12521407; doi:10.1038/s41467-025-63972-4)
Supplement: Supplementary file 3 — Reporting Summary [file 41467_2025_63972_MOESM3_ESM.pdf]

nature portfolio

Corresponding author(s): Seth Seegobin

Last updated by author(s): Jul 22, 2025

Reporting Summary

Nature Portfolio wishes to improve the reproducibility of the work that we publish. This form provides structure for consistency and transparency in reporting. For further information on Nature Portfolio policies, see our Editorial Policies and the Editorial Policy Checklist.

Statistics

For all statistical analyses, confirm that the following items are present in the figure legend, table legend, main text, or Methods section.

n/a	Confirmed	
☐	☒	The exact sample size (*n*) for each experimental group/condition, given as a discrete number and unit of measurement
☒	☐	A statement on whether measurements were taken from distinct samples or whether the same sample was measured repeatedly
☐	☒	The statistical test(s) used AND whether they are one- or two-sided<br>*Only common tests should be described solely by name; describe more complex techniques in the Methods section.*
☐	☒	A description of all covariates tested
☒	☐	A description of any assumptions or corrections, such as tests of normality and adjustment for multiple comparisons
☐	☒	A full description of the statistical parameters including central tendency (e.g. means) or other basic estimates (e.g. regression coefficient) AND variation (e.g. standard deviation) or associated estimates of uncertainty (e.g. confidence intervals)
☐	☒	For null hypothesis testing, the test statistic (e.g. *F*, *t*, *r*) with confidence intervals, effect sizes, degrees of freedom and *P* value noted<br>*Give P values as exact values whenever suitable.*
☒	☐	For Bayesian analysis, information on the choice of priors and Markov chain Monte Carlo settings
☒	☐	For hierarchical and complex designs, identification of the appropriate level for tests and full reporting of outcomes
☒	☐	Estimates of effect sizes (e.g. Cohen's *d*, Pearson's *r*), indicating how they were calculated

Our web collection on statistics for biologists contains articles on many of the points above.

Software and code

Policy information about availability of computer code

Data collection	Not applicable
Data analysis	All analyses were conducted using the commercially available SAS version 9.4 or higher (SAS Institute, Cary, NC, USA). Custom code used for fitting the ToP model described in this manuscript as well as a sample dataset for running the code may be obtained from the Github repository at https://github.com/AstraZeneca/ToPModel/

For manuscripts utilizing custom algorithms or software that are central to the research but not yet described in published literature, software must be made available to editors and reviewers. We strongly encourage code deposition in a community repository (e.g. GitHub). See the Nature Portfolio guidelines for submitting code & software for further information.

Data

Policy information about availability of data

All manuscripts must include a data availability statement. This statement should provide the following information, where applicable:

- Accession codes, unique identifiers, or web links for publicly available datasets
- A description of any restrictions on data availability
- For clinical datasets or third party data, please ensure that the statement adheres to our policy

The data underlying the findings described in this manuscript consist of anonymized patient-level datasets from clinical trials, which cannot be freely shared to protect patient privacy in accordance with General Data Protection Regulation (GDPR) and other applicable local legislation, and subject to the content of informed consent forms, access can be obtained in accordance with AstraZeneca's data sharing policy described at https://www.astrazenecaclinicaltrials.com/our-

transparency-commitments/. Data for studies directly listed on Vivli can be requested through Vivli at www.vivli.org. Data for studies not listed on Vivli could be requested through Vivli at https://vivli.org/members/enquiries-about-studies-not-listed-on-the-vivli-platform/. AstraZeneca Vivli member page is also available outlining further details: https://vivli.org/ourmember/astrazeneca. Data will be made available upon approval of the request and signature of the Data Usage Agreement, Typically, data will be available up to one year starting on the date access was granted.

Research involving human participants, their data, or biological material

Policy information about studies with human participants or human data. See also policy information about sex, gender (identity/presentation), and sexual orientation and race, ethnicity and racism.

Reporting on sex and gender	Sex of the participants in the studies that provided data for this analysis were reported in the primary trial reports referenced in the manuscript (PROVENT: Levin et al, 2022: https://www.nejm.org/doi/full/10.1056/NEJMoa2116620, SUPERNOVA: Haidar et al 2025; https://linkinghub.elsevier.com/retrieve/pii/S1473-3099(24)00804-1)
Reporting on race, ethnicity, or other socially relevant groupings	Race and ethnicity of participants in the studies that provided data for this analysis were reported in the primary trial reports referenced in the manuscript.
Population characteristics	Baseline characteristics of the participants in the studies that provided data for this analysis were reported in the primary trial reports referenced in the manuscript.
Recruitment	Details of participant recruitment in the studies that provided data for this analysis were reported in the primary trial reports referenced in the manuscript.
Ethics oversight	We have included the list of IRBs/ECs that approved the studies referenced in the manuscript (Supplementary Data 1 and 2)

Note that full information on the approval of the study protocol must also be provided in the manuscript.

Field-specific reporting

Please select the one below that is the best fit for your research. If you are not sure, read the appropriate sections before making your selection.

[x] Life sciences [ ] Behavioural & social sciences [ ] Ecological, evolutionary & environmental sciences

For a reference copy of the document with all sections, see nature.com/documents/nr-reporting-summary-flat.pdf

Life sciences study design

All studies must disclose on these points even when the disclosure is negative.

Sample size	Sample size was based on available data from the PROVENT and SUPERNOVA study. Sample size for PROVENT is described in Levin et al 2022 (https://www.nejm.org/doi/full/10.1056/NEJMoa2116620), and for SUPERNOVA in Haidar et al 2025 (https://linkinghub.elsevier.com/retrieve/pii/S1473-3099(24)00804-1)
Data exclusions	mAb serum concentrations were excluded if not quantifiable
Replication	Serum mAb concentrations were generated using popPK models. The output was the mean of 10 replicates per individual, performed by sampling from between-subject variability. Further details are included in the supplementary appendix.
Randomization	Participants were centrally assigned to active treatment or comparator using interactive response technology (IRT). This is described in the primary trial reports as referenced above.
Blinding	All participants and investigators involved in the dosing, clinical evaluation, and monitoring of the participants were blinded to which randomized drug was received. This is described in the primary trial reports referenced above.

Reporting for specific materials, systems and methods

We require information from authors about some types of materials, experimental systems and methods used in many studies. Here, indicate whether each material, system or method listed is relevant to your study. If you are not sure if a list item applies to your research, read the appropriate section before selecting a response.

Materials & experimental systems

n/a	Involved in the study
☒	☐ Antibodies
☒	☐ Eukaryotic cell lines
☒	☐ Palaeontology and archaeology
☒	☐ Animals and other organisms
☐	☒ Clinical data
☒	☐ Dual use research of concern
☒	☐ Plants

Methods

n/a	Involved in the study
☒	☐ ChIP-seq
☒	☐ Flow cytometry
☒	☐ MRI-based neuroimaging

Clinical data

Policy information about clinical studies

All manuscripts should comply with the ICMJEguidelines for publication of clinical research and a completedCONSORT checklist must be included with all submissions.

Clinical trial registration	NCT04625725, NCT05648110
Study protocol	Available with primary publications for Levin et al and Haidar et al (see links above)
Data collection	Study recruitment for PROVENT took place across five countries (Belgium, France, Spain, UK and the USA) between November 2020 and March 2021. Recruitment for SUPERNOVA took place across 18 countries between March and October 2023.
Outcomes	Primary outcomes in both studies was incidence of symptomatic COVID-19 with active treatment compared with comparator in participants with negative baseline SARS-CoV-2 RT-PCR test

Plants

Seed stocks	Not applicable
Novel plant genotypes	Not applicable
Authentication	Not applicable
