# Supplementary material for: A SARS-CoV-2 variant‑adjusted threshold of protection model for monoclonal antibody pre-exposure prophylaxis against COVID-19
Source: Nat Commun. 2025 Oct 14;16:9101. doi: 10.1038/s41467-025-63972-4 (PMC12521407; doi:10.1038/s41467-025-63972-4)
Supplement: Supplementary file 4 — Supplementary file [file 41467_2025_63972_MOESM4_ESM.pdf]

Supplementary data 1: Independent ethics committees/institutional review boards, and informed consent form approval for PROVENT

Supplementary data 2: Independent ethics committees/institutional review boards, and informed consent form approval for SUPERNOVA
