# Supplementary material for: A SARS-CoV-2 variant‑adjusted threshold of protection model for monoclonal antibody pre-exposure prophylaxis against COVID-19
Source: Nat Commun. 2025 Oct 14;16:9101. doi: 10.1038/s41467-025-63972-4 (PMC12521407; doi:10.1038/s41467-025-63972-4)
Supplement: Supplementary file 5 — Supplementary Data1 [file 41467_2025_63972_MOESM5_ESM.pdf]

Clinical Study Report Appendix 16.1.3

Drug Substance	AZD7442
Study Code	D8850C00002

Appendix 16.1.3
Independent Ethics Committees/Institutional Review Boards Consulted, and Samples of Written Subject Information and Consent Form

16.1.3.1 Independent Ethics Committees/Institutional Review Boards consulted

Country	Centre no.	Name and address of IEC/IRB	Chairman of IEC/IRB	Date of Approval
Belgium	0501	CHU UCL Namur, site Godinne Comité d’éthique, Avenue Docteur G. Thérasse 1, 5530 YVOIR	Prof. Patrick Evrard	25-Nov-2020
Belgium	0502	CHU UCL Namur, site Godinne Comité d’éthique, Avenue Docteur G. Thérasse 1, 5530 YVOIR	Prof. Patrick Evrard	25-Nov-2020
Belgium	0503	CHU UCL Namur, site Godinne Comité d’éthique, Avenue Docteur G. Thérasse 1, 5530 YVOIR	Prof. Patrick Evrard	25-Nov-2020
Belgium	0504	CHU UCL Namur, site Godinne Comité d’éthique, Avenue Docteur G. Thérasse 1, 5530 YVOIR	Prof. Patrick Evrard	25-Nov-2020
Belgium	0505	CHU UCL Namur, site Godinne Comité d’éthique, Avenue Docteur G. Thérasse 1, 5530 YVOIR	Prof. Patrick Evrard	25-Nov-2020
France	2301	Comite De Protection Des Personnes- Ile de France III, 89 rue d'Assas, 75006 Paris	Pierre Loulergue	08-Dec-2020
France	2302	Comite De Protection Des Personnes- Ile de France III, 89 rue d'Assas, 75006 Paris	Pierre Loulergue	08-Dec-2020
France	2304	Comite De Protection Des Personnes- Ile de France III, 89 rue d'Assas, 75006 Paris	Pierre Loulergue	08-Dec-2020

Country	Centre no.	Name and address of IEC/IRB	Chairman of IEC/IRB	Date of Approval
France	2305	Comite De Protection Des Personnes- Ile de France III, 89 rue d'Assas, 75006 Paris	Pierre Loulergue	08-Dec-2020
France	2306	Comite De Protection Des Personnes- Ile de France III, 89 rue d'Assas, 75006 Paris	Pierre Loulergue	08-Dec-2020
France	2308	Comite De Protection Des Personnes- Ile de France III, 89 rue d'Assas, 75006 Paris	Pierre Loulergue	08-Dec-2020
France	2309	Comite De Protection Des Personnes- Ile de France III, 89 rue d'Assas, 75006 Paris	Pierre Loulergue	08-Dec-2020
France	2310	Comite De Protection Des Personnes- Ile de France III, 89 rue d'Assas, 75006 Paris	Pierre Loulergue	08-Dec-2020
France	2311	Comite De Protection Des Personnes- Ile de France III, 89 rue d'Assas, 75006 Paris	Pierre Loulergue	08-Dec-2020
France	2312	Comite De Protection Des Personnes- Ile de France III, 89 rue d'Assas, 75006 Paris	Pierre Loulergue	08-Dec-2020
Spain	7001	Celm Hospital, Clinic Barcelona C/ Villarroel 170, 8036 Barcelona	Joaquim Forés I Viñeta	13-Jan-2021
Spain	7002	Celm Hospital, Clinic Barcelona C/ Villarroel 170, 8036 Barcelona	Joaquim Forés I Viñeta	13-Jan-2021
Spain	7003	Celm Hospital, Clinic Barcelona C/ Villarroel 170, 8036 Barcelona	Joaquim Forés I Viñeta	13-Jan-2021
Spain	7004	Celm Hospital, Clinic Barcelona C/ Villarroel 170, 8036 Barcelona	Joaquim Forés I Viñeta	13-Jan-2021
Spain	7005	Celm Hospital, Clinic Barcelona C/ Villarroel 170, 8036 Barcelona	Joaquim Forés I Viñeta	13-Jan-2021

Country	Centre no.	Name and address of IEC/IRB	Chairman of IEC/IRB	Date of Approval
United Kingdom	2810	London- Fulham Research Ethics Committee, Barlow House 3rd Floor, 4 Minshull Street, M1 3DZ Manchester	Reverend Nigel Griffin	18-Nov-2020
United Kingdom	2811	London- Fulham Research Ethics Committee, Barlow House 3rd Floor, 4 Minshull Street, M1 3DZ Manchester	Reverend Nigel Griffin	18-Nov-2020
United Kingdom	2812	London- Fulham Research Ethics Committee, Barlow House 3rd Floor, 4 Minshull Street, M1 3DZ Manchester	Reverend Nigel Griffin	18-Nov-2020
United Kingdom	2813	London- Fulham Research Ethics Committee, Barlow House 3rd Floor, 4 Minshull Street, M1 3DZ Manchester	Reverend Nigel Griffin	18-Nov-2020
United Kingdom	2814	London- Fulham Research Ethics Committee, Barlow House 3rd Floor, 4 Minshull Street, M1 3DZ Manchester	Reverend Nigel Griffin	18-Nov-2020
United Kingdom	2815	London- Fulham Research Ethics Committee, Barlow House 3rd Floor, 4 Minshull Street, M1 3DZ Manchester	Reverend Nigel Griffin	18-Nov-2020
United Kingdom	2816	London- Fulham Research Ethics Committee, Barlow House 3rd Floor, 4 Minshull Street, M1 3DZ Manchester	Reverend Nigel Griffin	18-Nov-2020

Country	Centre no.	Name and address of IEC/IRB	Chairman of IEC/IRB	Date of Approval
United Kingdom	2817	London- Fulham Research Ethics Committee, Barlow House 3rd Floor, 4 Minshull Street, M1 3DZ Manchester	Reverend Nigel Griffin	28-Oct-2020
United Kingdom	2818	London- Fulham Research Ethics Committee, Barlow House 3rd Floor, 4 Minshull Street, M1 3DZ Manchester	Reverend Nigel Griffin	28-Oct-2020
United States	7801	WIRB Copernicus, 5000 CentreGreen Way, 27513 NC Cary	Donald A. Deieso	22-Dec-2020
United States	7802	WIRB Copernicus, 5000 CentreGreen Way, 27513 NC Cary	Donald A. Deieso	25-Nov-2020
United States	7804	WIRB Copernicus, 5000 CentreGreen Way, 27513 NC Cary	Donald A. Deieso	22-Dec-2020
United States	7806	WIRB Copernicus, 5000 CentreGreen Way, 27513 NC Cary	Donald A. Deieso	25-Nov-2020
United States	7807	WIRB Copernicus, 5000 CentreGreen Way, 27513 NC Cary	Donald A. Deieso	25-Nov-2020
United States	7809	WIRB Copernicus, 5000 CentreGreen Way, 27513 NC Cary	Donald A. Deieso	01-Dec-2020
United States	7810	WIRB Copernicus, 5000 CentreGreen Way, 27513 NC Cary	Donald A. Deieso	17-Dec-2020
United States	7813	WIRB Copernicus, 5000 CentreGreen Way, 27513 NC Cary	Donald A. Deieso	22-Dec-2020
United States	7816	WIRB Copernicus, 5000 CentreGreen Way, 27513 NC Cary	Donald A. Deieso	02-Dec-2020
United States	7818	WIRB Copernicus, 5000 CentreGreen Way, 27513 NC Cary	Donald A. Deieso	30-Nov-2020

Country	Centre no.	Name and address of IEC/IRB	Chairman of IEC/IRB	Date of Approval
United States	7819	WIRB Copernicus, 5000 CentreGreen Way, 27513 NC Cary	Donald A. Deieso	18-Dec-2020
United States	7820	WIRB Copernicus, 5000 CentreGreen Way, 27513 NC Cary	Donald A. Deieso	17-Dec-2020
United States	7822	WIRB Copernicus, 5000 CentreGreen Way, 27513 NC Cary	Donald A. Deieso	08-Jan-2021
United States	7823	WIRB Copernicus, 5000 CentreGreen Way, 27513 NC Cary	Donald A. Deieso	01-Dec-2020
United States	7824	WIRB Copernicus, 5000 CentreGreen Way, 27513 NC Cary	Donald A. Deieso	30-Nov-2020
United States	7825	WIRB Copernicus, 5000 CentreGreen Way, 27513 NC Cary	Donald A. Deieso	17-Dec-2020
United States	7826	WIRB Copernicus, 5000 CentreGreen Way, 27513 NC Cary	Donald A. Deieso	01-Dec-2020
United States	7827	WIRB Copernicus, 5000 CentreGreen Way, 27513 NC Cary	Donald A. Deieso	22-Dec-2020
United States	7828	WIRB Copernicus, 5000 CentreGreen Way, 27513 NC Cary	Donald A. Deieso	18-Dec-2020
United States	7830	WIRB Copernicus, 5000 CentreGreen Way, 27513 NC Cary	Donald A. Deieso	30-Nov-2020
United States	7831	WIRB Copernicus, 5000 CentreGreen Way, 27513 NC Cary	Donald A. Deieso	22-Dec-2020
United States	7832	WIRB Copernicus, 5000 CentreGreen Way, 27513 NC Cary	Donald A. Deieso	23-Dec-2020
United States	7833	WIRB Copernicus, 5000 CentreGreen Way, 27513 NC Cary	Donald A. Deieso	30-Nov-2020
United States	7835	WIRB Copernicus, 5000 CentreGreen Way, 27513 NC Cary	Donald A. Deieso	17-Dec-2020

Country	Centre no.	Name and address of IEC/IRB	Chairman of IEC/IRB	Date of Approval
United States	7836	WIRB Copernicus, 5000 CentreGreen Way, 27513 NC Cary	Donald A. Deieso	25-Nov-2020
United States	7838	WIRB Copernicus, 5000 CentreGreen Way, 27513 NC Cary	Donald A. Deieso	01-Dec-2020
United States	7839	WIRB Copernicus, 5000 CentreGreen Way, 27513 NC Cary	Donald A. Deieso	24-Nov-2020
United States	7840	WIRB Copernicus, 5000 CentreGreen Way, 27513 NC Cary	Donald A. Deieso	30-Nov-2020
United States	7841	WIRB Copernicus, 5000 CentreGreen Way, 27513 NC Cary	Donald A. Deieso	18-Dec-2020
United States	7843	WIRB Copernicus, 5000 CentreGreen Way, 27513 NC Cary	Donald A. Deieso	18-Dec-2020
United States	7844	WIRB Copernicus, 5000 CentreGreen Way, 27513 NC Cary	Donald A. Deieso	18-Dec-2020
United States	7847	WIRB Copernicus, 5000 CentreGreen Way, 27513 NC Cary	Donald A. Deieso	24-Nov-2020
United States	7849	WIRB Copernicus, 5000 CentreGreen Way, 27513 NC Cary	Donald A. Deieso	25-Nov-2020
United States	7850	WIRB Copernicus, 5000 CentreGreen Way, 27513 NC Cary	Donald A. Deieso	25-Nov-2020
United States	7851	WIRB Copernicus, 5000 CentreGreen Way, 27513 NC Cary	Donald A. Deieso	25-Nov-2020
United States	7852	WIRB Copernicus, 5000 CentreGreen Way, 27513 NC Cary	Donald A. Deieso	25-Nov-2020
United States	7853	WIRB Copernicus, 5000 CentreGreen Way, 27513 NC Cary	Donald A. Deieso	17-Dec-2020
United States	7854	WIRB Copernicus, 5000 CentreGreen Way, 27513 NC Cary	Donald A. Deieso	30-Nov-2020

Country	Centre no.	Name and address of IEC/IRB	Chairman of IEC/IRB	Date of Approval
United States	7855	WIRB Copernicus, 5000 CentreGreen Way, 27513 NC Cary	Donald A. Deieso	17-Dec-2020
United States	7856	WIRB Copernicus, 5000 CentreGreen Way, 27513 NC Cary	Donald A. Deieso	17-Dec-2020
United States	7857	WIRB Copernicus, 5000 CentreGreen Way, 27513 NC Cary	Donald A. Deieso	24-Nov-2020
United States	7859	WIRB Copernicus, 5000 CentreGreen Way, 27513 NC Cary	Donald A. Deieso	30-Nov-2020
United States	7860	WIRB Copernicus, 5000 CentreGreen Way, 27513 NC Cary	Donald A. Deieso	07-Dec-2020
United States	7861	WIRB Copernicus, 5000 CentreGreen Way, 27513 NC Cary	Donald A. Deieso	12-Jan-2021
United States	7863	WIRB Copernicus, 5000 CentreGreen Way, 27513 NC Cary	Donald A. Deieso	14-Jan-2021
United States	7864	WIRB Copernicus, 5000 CentreGreen Way, 27513 NC Cary	Donald A. Deieso	14-Jan-2021
United States	7865	WIRB Copernicus, 5000 CentreGreen Way, 27513 NC Cary	Donald A. Deieso	13-Jan-2021
United States	7866	WIRB Copernicus, 5000 CentreGreen Way, 27513 NC Cary	Donald A. Deieso	12-Jan-2021
United States	7868	WIRB Copernicus, 5000 CentreGreen Way, 27513 NC Cary	Donald A. Deieso	12-Jan-2021
United States	7870	WIRB Copernicus, 5000 CentreGreen Way, 27513 NC Cary	Donald A. Deieso	08-Jan-2021
United States	7873	WIRB Copernicus, 5000 CentreGreen Way, 27513 NC Cary	Donald A. Deieso	12-Jan-2021
United States	7874	WIRB Copernicus, 5000 CentreGreen Way, 27513 NC Cary	Donald A. Deieso	14-Jan-2021

Country	Centre no.	Name and address of IEC/IRB	Chairman of IEC/IRB	Date of Approval
United States	7877	WIRB Copernicus, 5000 CentreGreen Way, 27513 NC Cary	Donald A. Deieso	14-Jan-2021
United States	7878	WIRB Copernicus, 5000 CentreGreen Way, 27513 NC Cary	Donald A. Deieso	11-Jan-2021
United States	7879	WIRB Copernicus, 5000 CentreGreen Way, 27513 NC Cary	Donald A. Deieso	05-Jan-2021
United States	7880	WIRB Copernicus, 5000 CentreGreen Way, 27513 NC Cary	Donald A. Deieso	30-Nov-2020
United States	7881	WIRB Copernicus, 5000 CentreGreen Way, 27513 NC Cary	Donald A. Deieso	24-Nov-2020
United States	7883	WIRB Copernicus, 5000 CentreGreen Way, 27513 NC Cary	Donald A. Deieso	30-Nov-2020

16.1.3.2 Samples of written Subject Information and Consent Form

Global ICF Name and Version No	Version Date
Adult Study Subject Master Information and Consent Form v.1.0	06-Oct-2020
Adult Study Information and Consent Form for Pregnant Partners of Study Subjects v.1.0	09-Oct-2020
Adult Study Subject Master Information and Consent Form v. 2.0	12-Nov-2020
Study Subject Assent Form v.1.0	09-Nov-2020
Adult Study Subject Master Information and Consent Form v.3.0	21-Dec-2020
Study Subject Assent Form v.2.0	21-Dec-2020
Adult Study Subject Master Information and Consent Form v.4.0	12-Feb-2021
Adult Study Information and Consent Form for Pregnant Partners of Study Subjects v.2.0	16-Feb-2021
Adult Study Subject Master Information and Consent Form v.5.0	17-Mar-2021
Study Subject Assent Form v.3.0	17-Mar-2021
Adult Study Subject Master Information and Consent Form v.6.0	02-Dec-2021
Adult Study Subject Master Information and Consent Form **Addendum** v.1.0	02-Dec-2021
Study Subject Assent Form **Addendum** v.1.0	02-Dec-2021
