# Supplementary material for: A SARS-CoV-2 variant‑adjusted threshold of protection model for monoclonal antibody pre-exposure prophylaxis against COVID-19
Source: Nat Commun. 2025 Oct 14;16:9101. doi: 10.1038/s41467-025-63972-4 (PMC12521407; doi:10.1038/s41467-025-63972-4)
Supplement: Supplementary file 6 — Supplementary Data 2 [file 41467_2025_63972_MOESM6_ESM.pdf]

**Clinical Study Report Appendix 16.1.3**	
Drug Substance	Sipavibart
Study Code	D7000C00001

Appendix 16.1.3
Independent Ethics Committees/Institutional Review Boards Consulted, and Informed Consent Form

16.1.3.1 Independent Ethics Committees/Institutional Review Boards consulted

Country	Site no.	Name and address of IEC/IRB	Chairman of IEC/IRB	Date of Approval
Australia	301	Mater Misericordiae Limited Human Research Ethics committee RGO 76 Willetts Rd,North Mackay,Queensland,4740	Professor J McNeil	07-Jun-23
Australia	302	Alfred Hospital Ethics Committee (RGO) 55 Commercial Road,The Alfred Clinical Trials Pharmacy,Melbourne,Victoria,3004	Brian Stoffell	18-Aug-23
Australia	304	Peter MacCallum Cancer Centre Ethics Committee (RGO) Level 4, 305 Grattan Street,East Melbourne,Victoria,3002	Arnau Garcia-Clapes	10-Aug-23
Australia	305	South Metropolitan Health Service Local Ethics Committee 11 Robin Warren Drive,,Murdoch,Western Australia,6150	Melanie Wright	16-Aug-23
Australia	306	Perth Blood Institute 18 Prowse Street,West Perth,Western Australia,6005	David Sneddon	31-May-23
Australia	307	University Sunshine Coast 90 Sippy Down Road,Sippy Downs,Queensland,4556	Angela Bestard	12-Jul-23
Australia	308	Melbourne Health. The Royal Melbourne Hospital , City Campus, 300 Grattan Street,,Parkville,Victoria, 3050	Dr Jacqui Waterkeyn	31-Jul-23
Belgium	501	Level 3, Block B	Prof. Dr. Filip Cools	21-Jun-23

Country	Site no.	Name and address of IEC/IRB	Chairman of IEC/IRB	Date of Approval
Belgium	502	CHU de Liège Domaine Universitaire du Sart Tilman,Batiment B 35,Liege,B-4000	V. Seutin	30-Jun-23
Canada	1001	Comité d'éthique de la recherche du CHUM 900, rue St-Denis,Tour Viger, 3e étage, Salle R03-216,Montréal,Quebec,H2X 0A9	Antonine Pouyes Rigaud	07-Sep-23
Canada	1008	Comité d'éthique de la recherche du CHUM 900, rue St-Denis,Tour Viger, 3e étage, Salle R03-216,Montréal,Quebec,H2X 0A9	Kassandy Kowalyk	22-Sep-23
Denmark	2001	The Medical Ethics Committees (VMK) Ørestads Boulevard 5 2300 Copenhagen Denmark	Tarec Christoffer EL-Galaly	08-May-23
Denmark	2002	The Medical Ethics Committees (VMK) Ørestads Boulevard 5 2300 Copenhagen Denmark	Tarec Christoffer EL-Galaly	08-May-23
Denmark	2003	The Medical Ethics Committees (VMK) Ørestads Boulevard 5 2300 Copenhagen Denmark	Tarec Christoffer EL-Galaly	26Jul2023
Denmark	2005	The Medical Ethics Committees (VMK) Ørestads Boulevard 5 2300 Copenhagen Denmark	Tarec Christoffer EL-Galaly	08-May-23
Denmark	2006	The Medical Ethics Committees (VMK) Ørestads Boulevard 5 2300 Copenhagen Denmark	Tarec Christoffer EL-Galaly	13Jan2023

Country	Site no.	Name and address of IEC/IRB	Chairman of IEC/IRB	Date of Approval
France	2301	the Comité de protection des personnes Sud-Est II	Ms. Carine Ursini-Maurin	19-Jul-23
France	2302	the Comité de protection des personnes Sud-Est II	Ms. Carine Ursini-Maurin	19-Jul-23
France	2305	the Comité de protection des personnes Sud-Est II	Ms. Carine Ursini-Maurin	19-Jul-23
France	2307	the Comité de protection des personnes Sud-Est II	Ms. Carine Ursini-Maurin	19-Jul-23
France	2310	the Comité de protection des personnes Sud-Est II	Ms. Carine Ursini-Maurin	19-Jul-23
France	2403	the Comité de protection des personnes Sud-Est II	Ms. Carine Ursini-Maurin	19-Jul-23
France	2406	the Comité de protection des personnes Sud-Est II	Ms. Carine Ursini-Maurin	19-Jul-23
France	2408	the Comité de protection des personnes Sud-Est II	Ms. Carine Ursini-Maurin	19-Jul-23
France	2409	the Comité de protection des personnes Sud-Est II	Ms. Carine Ursini-Maurin	19-Jul-23
France	2410	the Comité de protection des personnes Sud-Est II	Ms. Carine Ursini-Maurin	19-Jul-23
France	2412	the Comité de protection des personnes Sud-Est II	Ms. Carine Ursini-Maurin	19-Jul-23
France	2413	the Comité de protection des personnes Sud-Est II	Ms. Carine Ursini-Maurin	19-Jul-23
Germany	2701	Ärztekammer Nordrhein Ethikkommission Tersteegenstr. 9 40474 Düsseldorf Deutschland	Prof. Dr. Kurt Rackè	26Jul2023
Germany	2702	Ärztekammer Hamburg Ethikkommission Weidestraße 122 b 22083 Hamburg Deutschland	Prof. Dr. Kurt Rackè	12-May-23
Germany	2703	Geschäftsstelle der Ethikkommission der Universität zu Köln Kerpener Str. 62 50937 Köln Deutschland	Prof. Dr. Kurt Rackè	12-Oct-23

Country	Site no.	Name and address of IEC/IRB	Chairman of IEC/IRB	Date of Approval
Germany	2706	Ethik-Kommission der Medizinische Hochschule Hannover<br>Sekretariat der Ethikkommission<br>- OE 9515 -<br>Carl-Neuberg-Str. 1<br>30625 Hannover<br>Deutschland	Prof. Dr. Kurt Rackè	12-May-23
Germany	2707	Landesärztekammer Rheinland-Pfalz<br>Ethikkommission<br>Deutschhausplatz 3<br>55116 Mainz<br>Deutschland	Prof. Dr. Kurt Rackè	12-May-23
Germany	2709	Ethik-Kommission der Medizinischen Fakultät der Universität Duisburg-Essen<br>Universitätsklinikum Essen<br>Robert-Koch-Str. 9-11<br>45147 Essen<br>Deutschland	Prof. Dr. Kurt Rackè	23-Jun-23
United Kingdom	2801	London - Harrow Research Ethics Committee<br>Level 3, Block B<br>Whitefriars<br>Lewins Mead<br>Bristol<br>BS1 2NT	Dr. Linda McDonald	13-Dec-22
United Kingdom	2802	London - Harrow Research Ethics Committee<br>Level 3, Block B<br>Whitefriars<br>Lewins Mead<br>Bristol<br>BS1 2NT	Dr. Linda McDonald	13-Dec-22

Country	Site no.	Name and address of IEC/IRB	Chairman of IEC/IRB	Date of Approval
United Kingdom	2803	London - Harrow Research Ethics Committee Level 3, Block B Whitefriars Lewins Mead Bristol BS1 2NT	Dr. Linda McDonald	13-Dec-22
United Kingdom	2804	London - Harrow Research Ethics Committee Level 3, Block B Whitefriars Lewins Mead Bristol BS1 2NT	Dr. Linda McDonald	13-Dec-22
United Kingdom	2805	London - Harrow Research Ethics Committee Level 3, Block B Whitefriars Lewins Mead Bristol BS1 2NT	Dr. Linda McDonald	13-Dec-22
United Kingdom	2806	R&D UCLH NHS Foundation Trust Joint Research Office,1st Floor Maple House Suite B,149 Tottenham Court Road,London,Greater London,W1T 7DN	Dr Linda McDonald	22-Feb-23
United Kingdom	2807	London - Harrow Research Ethics Committee Level 3, Block B Whitefriars Lewins Mead Bristol BS1 2NT	Dr. Linda McDonald	13-Dec-22

Country	Site no.	Name and address of IEC/IRB	Chairman of IEC/IRB	Date of Approval
United Kingdom	2808	London - Harrow Research Ethics Committee<br>Level 3, Block B<br>Whitefriars<br>Lewins Mead<br>Bristol<br>BS1 2NT	Dr. Linda McDonald	13-Dec-22
United Kingdom	2809	London - Harrow Research Ethics Committee<br>Level 3, Block B<br>Whitefriars<br>Lewins Mead<br>Bristol<br>BS1 2NT	Dr. Linda McDonald	13-Dec-22
United Kingdom	2810	London - Harrow Research Ethics Committee<br>Level 3, Block B<br>Whitefriars<br>Lewins Mead<br>Bristol<br>BS1 2NT	Dr. Linda McDonald	13-Dec-22
United Kingdom	2812	London - Harrow Research Ethics Committee<br>Level 3, Block B<br>Whitefriars<br>Lewins Mead<br>Bristol<br>BS1 2NT	Dr. Linda McDonald	03-May-23
United Kingdom	2814	London - Harrow Research Ethics Committee<br>Level 3, Block B<br>Whitefriars<br>Lewins Mead<br>Bristol<br>BS1 2NT	Dr. Linda McDonald	13-Dec-22
Israel	4001	Chaim Sheba MC Ethics Committee<br>Tel Hashomer,Ramat Gan,5265602	Prof. Merav Lidar	18-Jul-23

Country	Site no.	Name and address of IEC/IRB	Chairman of IEC/IRB	Date of Approval
Israel	4003	Rabin MC Ethics Committee<br>39 Jabotinsky St Ground floor,Beilinson Campus Raphael,Recanati Genetic Inst. Blg,Gaucher Clinic, Genetics Institute,Petach Tikva,4941492	Prof. Ran Tur-Kaspa<br>Helsinki Committee Chairperson<br>Rabin Medical Center	30-Jul-23
Malaysia	4801	MREC	Dr. Nurain Binti Mohd Noor	13Jan2023
Malaysia	4802	MREC	Dr. Nurain Binti Mohd Noor	09-Jun-23
Malaysia	4803	Pusat Perubatan Universiti Kebangsaan Malaysia<br>Makmal Pemeriksaan (Makmal Endokrin),Cheras,Makmal Pemeriksaan (Makmal Endokrin),Kuala Lumpur,Kuala Lumpur,56000	DR. Mohd Shahrir Mohamed Said	16-Jun-23
Malaysia	4804	Medical Research Ethics Committee (MREC UMMC)<br>Lembah Pantai,Medical,Kuala Lumpur,Kuala Lumpur,59100	DR. NIK SHERINA HAIDI BT HANAFI	11-Aug-23
Malaysia	4805	Sunway Medical Centre Independent Research Ethics Committee<br>Sunway Medical Centre,No. 5 Jalan Lagoon Selatan,Bandar Sunway,Petaling Jaya,Selangor,46150	Dr. Quek Kia Fatt	27-Jun-23
Poland	5702	Komisja Bioetyczna przy Okręgowej Izbie Lekarskiej w Lublinie<br>Ul. Chmielna 4, 20-079 Lublin	Dr n. med. Janusz Dubejko	26-Sep-23

Country	Site no.	Name and address of IEC/IRB	Chairman of IEC/IRB	Date of Approval
Poland	5704	Komisja Bioetyczna przy Okręgowej Izbie Lekarskiej w Lublinie Ul. Chmielna 4, 20-079 Lublin	Dr n. med. Janusz Dubejko	26-Sep-23
Korea, Republic of	6001	IRB of Seoul National University Hospital 101 Daehak-ro Jongno-gu,Seoul,Not Applicable,3080	Hong Yun-Chul;<br>Yoon Jun Kim;<br>Chul-Gyu Yoo;<br>Gi Jeong Cheong;<br>Whal Lee;<br>Yong-Min Ahn	23-Dec-22
Korea, Republic of	6002	IRB of Seoul National University Bundang Hospital 82, Gumi-ro 173 beon-gil,Bundang-gu,Seongnam,Gyeonggi-do,KS009	Kyoung Un Park;<br>Hak Chul Jang;<br>Chang-Hun Huh;<br>Gheeyoung Choe;<br>Jin-Hyeok Hwang;<br>Chang-Hun Huh;<br>Suk Ki Cho	02-Jan-23
Korea, Republic of	6003	IRB of Asan Medical Center 88 Olympic-ro 43-gil, Songpa-gu,Clinical Trial Pharmacy, 5th floor, West Building,Seoul,Not Applicable,5505	Min-Hee Ryu	12-Dec-22
Korea, Republic of	6005	IRB of Korea University Guro Hospital 148, Gurodong-ro, Guro-gu,,Seoul,Not Applicable,8308	Not disclosed	20-Apr-23
Singapore	6601	NHG DSRB (Domain Specific Review Board) Fusionopolis Link #03-08 Nexus@one-north, Singapore 138543	A/Prof Madelynn Chan	04-Aug-23
Singapore	6602	NHG DSRB (Domain Specific Review Board) Fusionopolis Link #03-08 Nexus@one-north, Singapore 138543	A/Prof Madelynn Chan	04-Aug-23

Country	Site no.	Name and address of IEC/IRB	Chairman of IEC/IRB	Date of Approval
Singapore	6603	NHG DSRB (Domain Specific Review Board) Fusionopolis Link #03-08 Nexus@one-north, Singapore 138543	A/Prof Madelynn Chan	04-Aug-23
Spain	7001	CEIm Hospital General Universitario Gregorio Marañón	D. Andrés Jesús Muñoz Martín (Oncología Médica)	13-Jan-23
Spain	7002	CEIm Hospital General Universitario Gregorio Marañón	D. Andrés Jesús Muñoz Martín (Oncología Médica)	13-Jan-23
Spain	7004	Lewins Mead	D. Andrés Jesús Muñoz Martín (Oncología Médica)	13-Jan-23
Spain	7005	CEIm Hospital General Universitario Gregorio Marañón	D. Andrés Jesús Muñoz Martín (Oncología Médica)	26-Apr-23
Spain	7006	Whitefriars	D. Andrés Jesús Muñoz Martín (Oncología Médica)	26-Apr-23
Spain	7009	CEIm Hospital General Universitario Gregorio Marañón	D. Andrés Jesús Muñoz Martín (Oncología Médica)	26-Jul-23
Spain	7010	CEIm Hospital General Universitario Gregorio Marañón	D. Andrés Jesús Muñoz Martín (Oncología Médica)	26-Jul-23
Spain	7011	CEIm Hospital General Universitario Gregorio Marañón	D. Andrés Jesús Muñoz Martín (Oncología Médica)	26-Jul-23
Spain	7012	CEIm Hospital General Universitario Gregorio Marañón	D. Andrés Jesús Muñoz Martín (Oncología Médica)	26-Jul-23
Spain	7013	CEIm Hospital General Universitario Gregorio Marañón	D. Andrés Jesús Muñoz Martín (Oncología Médica)	26-Jul-23
Spain	7014	CEIm Hospital General Universitario Gregorio Marañón	D. Andrés Jesús Muñoz Martín (Oncología Médica)	26-Jul-23
Spain	7016	CEIm Hospital General Universitario Gregorio Marañón	D. Andrés Jesús Muñoz Martín (Oncología Médica)	26-Jul-23
Spain	7017	CEIm Hospital General Universitario Gregorio Marañón	D. Andrés Jesús Muñoz Martín (Oncología Médica)	26Jul2023

Country	Site no.	Name and address of IEC/IRB	Chairman of IEC/IRB	Date of Approval
Spain	7018	CEIm Hospital General Universitario Gregorio Marañón	D. Andrés Jesús Muñoz Martín (Oncología Médica)	27Sep2023
Taiwan, Republic of China	7403	China Medical University Hospital, Institutional Review Board 6th Meeting Room, B1, 1st Medical Buidling,No.2 Yuh-Der Road,,Taichung,40447	Martin M-T Fuh	02-May-23
Taiwan, Republic of China	7404	Tri-Service General Hospital, Institutional Review Board No.325, Sec. 2, Cheng-Kung Rd., Neihu,Taipei,11490	Yu Mu-Hsien	05-May-23
Thailand	7501	The Khon Kaen University Ethics Committee for Human Research 17th Floor, Room no. 1733 Somdetprasrinakarintra Building, Faculty of Medicine, Khon Kaen University,Nai Muang Sub-district,Muang,Khon Kaen,40002	Professor Ratanavadee Nanagara, M.D.	21-Aug-23
Thailand	7502	Siriraj Institutional Review Board (SiRB) His Majesty the King’s 80th Birthday Anniversary,5th December 2007 Building. 2nd Floor,,Room no. 210. 2 Prannok Road, Prannok,Bangkoknoi,Bangkok,10700	Narapom Prayoonwiwat	15-Jun-23

Country	Site no.	Name and address of IEC/IRB	Chairman of IEC/IRB	Date of Approval
Thailand	7503	Ethical Clearance Committee on Human Rights Related to Researches Involving Human Subjects<br>Ethics Research in Human Unit, 3rd Floor, Research and Welfares Building Room 360, Ramathibodi Hospital, Mahidol University,270 Rama VI Road,Ratchathewi,Bangkok,10400	Professor Chairat Shayakul, M.D	15-Aug-23
Thailand	7504	Research Ethics Committee, Faculty of Medicine, Chiangmai University<br>110 Intavaroros Road,Faculty of Medicine Chiangmai University,Muang,Chiang Mai,50200	Nipon Sermpanich	20-Jun-23
Thailand	7505	Research Ethics Committee, Faculty of Medicine, Chiangmai University<br>110 Intavaroros Road,Faculty of Medicine Chiangmai University,Muang,Chiang Mai,50200	Nipon Sermpanich	20-Jun-23
Thailand	7506	Institute Review Board of Bamrasnaradura Institute Infectious Diseases Institute<br>126 Tiwanont Road,Muang,Nonthaburi, 11000	Kritecho Siripassorn, M.D.	23-Aug-23
United States	7802	WCG IRB<br>212 Carnegie Center,Suite 301,Princeton,New Jersey,8540	Kelly FitzGerald, PhD, CIP	06-Apr-23

Country	Site no.	Name and address of IEC/IRB	Chairman of IEC/IRB	Date of Approval
United States	7803	WCG IRB<br>212 Carnegie Center,Suite 301,Princeton,New Jersey,8540	Kelly FitzGerald, PhD, CIP	16-Mar-23
United States	7805	WCG IRB<br>212 Carnegie Center,Suite 301,Princeton,New Jersey,8540	Kelly FitzGerald, PhD, CIP	15-Mar-23
United States	7806	WCG IRB<br>212 Carnegie Center,Suite 301,Princeton,New Jersey,8540	Kelly FitzGerald, PhD, CIP	15-Mar-23
United States	7807	WCG IRB<br>212 Carnegie Center,Suite 301,Princeton,New Jersey,8540	Kelly FitzGerald, PhD, CIP	17-Feb-23
United States	7808	WCG IRB<br>212 Carnegie Center,Suite 301,Princeton,New Jersey,8540	Kelly FitzGerald, PhD, CIP	16-Mar-23
United States	7809	WIRB<br>1019 39th Avenue SE,Suite 120,Puyallup,Washington ,98374	Kelly FitzGerald, PhD, CIP	30-Mar-23
United States	7810	WCG IRB<br>212 Carnegie Center,Suite 301,Princeton,New Jersey,8540	Kelly FitzGerald, PhD, CIP	03-Jul-23
United States	7811	WIRB<br>1019 39th Avenue SE,Suite 120,Puyallup,Washington ,98374	Kelly FitzGerald, PhD, CIP	17-Feb-23

Country	Site no.	Name and address of IEC/IRB	Chairman of IEC/IRB	Date of Approval
United States	7812	WIRB<br>1019 39th Avenue SE,Suite 120,Puyallup,Washington ,98374	Kelly FitzGerald, PhD, CIP	20-Mar-23
United States	7813	WCG IRB<br>212 Carnegie Center,Suite 301,Princeton,New Jersey,8540	Kelly FitzGerald, PhD, CIP	24-May-23
United States	7815	WCG IRB<br>212 Carnegie Center,Suite 301,Princeton,New Jersey,8540	Kelly FitzGerald, PhD, CIP	05-Apr-23
United States	7816	WCG IRB<br>212 Carnegie Center,Suite 301,Princeton,New Jersey,8540	Kelly FitzGerald, PhD, CIP	21-Mar-23
United States	7817	WCG IRB<br>212 Carnegie Center,Suite 301,Princeton,New Jersey,8540	Kelly FitzGerald, PhD, CIP	20-Mar-23
United States	7818	WCG IRB<br>212 Carnegie Center,Suite 301,Princeton,New Jersey,8540	Kelly FitzGerald, PhD, CIP	15-Mar-23
United States	7819	WCG IRB<br>212 Carnegie Center,Suite 301,Princeton,New Jersey,8540	Kelly FitzGerald, PhD, CIP	22-Mar-23
United States	7820	WCG IRB<br>212 Carnegie Center,Suite 301,Princeton,New Jersey,8540	Kelly FitzGerald, PhD, CIP	10-Mar-23

Country	Site no.	Name and address of IEC/IRB	Chairman of IEC/IRB	Date of Approval
United States	7821	WCG IRB 212 Carnegie Center,Suite 301,Princeton,New Jersey,8540	Kelly FitzGerald, PhD, CIP	30-Mar-23
United States	7822	WCG IRB 212 Carnegie Center,Suite 301,Princeton,New Jersey,8540	Kelly FitzGerald, PhD, CIP	30-Mar-23
United States	7823	WCG IRB 212 Carnegie Center,Suite 301,Princeton,New Jersey,8540	Kelly FitzGerald, PhD, CIP	27-Mar-23
United States	7824	WIRB 1019 39th Avenue SE,Suite 120,Puyallup,Washington ,98374	Kelly FitzGerald, PhD, CIP	18-Mar-23
United States	7825	WCG IRB 212 Carnegie Center,Suite 301,Princeton,New Jersey,8540	Kelly FitzGerald, PhD, CIP	10-Jul-23
United States	7826	WIRB 1019 39th Avenue SE,Suite 120,Puyallup,Washington ,98374	Kelly FitzGerald, PhD, CIP	18-Mar-23
United States	7827	WCG IRB 212 Carnegie Center,Suite 301,Princeton,New Jersey,8540	Kelly FitzGerald, PhD, CIP	17-Mar-23
United States	7828	WCG IRB 212 Carnegie Center,Suite 301,Princeton,New Jersey,8540	Kelly FitzGerald, PhD, CIP	16-Mar-23

Country	Site no.	Name and address of IEC/IRB	Chairman of IEC/IRB	Date of Approval
United States	7830	WCG IRB 212 Carnegie Center,Suite 301,Princeton,New Jersey,8540	Kelly FitzGerald, PhD, CIP	19-Apr-23
United States	7831	WCG IRB 212 Carnegie Center,Suite 301,Princeton,New Jersey,8540	Kelly FitzGerald, PhD, CIP	21-Mar-23
United States	7833	WCG IRB 212 Carnegie Center,Suite 301,Princeton,New Jersey,8540	Kelly FitzGerald, PhD, CIP	21-Jun-23
United States	7834	WCG IRB 212 Carnegie Center,Suite 301,Princeton,New Jersey,8540	Kelly FitzGerald, PhD, CIP	14-Apr-23
United States	7835	WCG IRB 212 Carnegie Center,Suite 301,Princeton,New Jersey,8540	Kelly FitzGerald, PhD, CIP	16-Mar-23
United States	7836	WCG IRB 212 Carnegie Center,Suite 301,Princeton,New Jersey,8540	Kelly FitzGerald, PhD, CIP	07-Jul-23
United States	7837	WCG IRB 212 Carnegie Center,Suite 301,Princeton,New Jersey,8540	Kelly FitzGerald, PhD, CIP	07-Jun-23
United States	7838	WCG IRB 212 Carnegie Center,Suite 301,Princeton,New Jersey,8540	Kelly FitzGerald, PhD, CIP	04-May-23

Country	Site no.	Name and address of IEC/IRB	Chairman of IEC/IRB	Date of Approval
United States	7839	WCG IRB<br>212 Carnegie Center,Suite 301,Princeton,New Jersey,8540	Kelly FitzGerald, PhD, CIP	06-Jul-23
United States	7840	WCG IRB<br>212 Carnegie Center,Suite 301,Princeton,New Jersey,8540	Kelly FitzGerald, PhD, CIP	10-May-23
United States	7841	WCG IRB<br>212 Carnegie Center,Suite 301,Princeton,New Jersey,8540	Kelly FitzGerald, PhD, CIP	29-Mar-23
United States	7842	WCG IRB<br>212 Carnegie Center,Suite 301,Princeton,New Jersey,8540	Kelly FitzGerald, PhD, CIP	16-Mar-23
United States	7843	WCG IRB<br>212 Carnegie Center,Suite 301,Princeton,New Jersey,8540	Kelly FitzGerald, PhD, CIP	11-Apr-23
United States	7844	Johns Hopkins Medicine Institutional Review Boards<br>8600 Old Georgetown Road,Research - 4th floor South Wing,Bethesda,Maryland ,20814	Richard Moore, MD	07-Jun-23
United States	7845	Duke University Health System Institutional Review Board<br>2200 West Main Street,Suite 900, Erwin Square,Campus Box # 104026, DUHS IRB,Durham,North Carolina,27705	Lantos Paul	18-Jul-23

Country	Site no.	Name and address of IEC/IRB	Chairman of IEC/IRB	Date of Approval
United States	7846	WCG IRB 212 Carnegie Center,Suite 301,Princeton,New Jersey,8540	Kelly FitzGerald, PhD, CIP	30-Mar-23
United States	7849	WCG IRB 212 Carnegie Center,Suite 301,Princeton,New Jersey,8540	Kelly FitzGerald, PhD, CIP	11-Apr-23
United States	7850	WCG IRB 212 Carnegie Center,Suite 301,Princeton,New Jersey,8540	Kelly FitzGerald, PhD, CIP	10-May-23
United States	7851	WCG IRB 212 Carnegie Center,Suite 301,Princeton,New Jersey,8540	Kelly FitzGerald, PhD, CIP	27-May-23
United States	7852	WCG IRB 212 Carnegie Center,Suite 301,Princeton,New Jersey,8540	Kelly FitzGerald, PhD, CIP	05-May-23
United States	7853	WCG IRB 212 Carnegie Center,Suite 301,Princeton,New Jersey,8540	Kelly FitzGerald, PhD, CIP	18-Jul-23
United States	7854	WCG IRB 212 Carnegie Center,Suite 301,Princeton,New Jersey,8540	Kelly FitzGerald, PhD, CIP	06-Apr-23
United States	7855	WCG IRB 212 Carnegie Center,Suite 301,Princeton,New Jersey,8540	Kelly FitzGerald, PhD, CIP	30-Mar-23

Country	Site no.	Name and address of IEC/IRB	Chairman of IEC/IRB	Date of Approval
United States	7856	WCG IRB 212 Carnegie Center,Suite 301,Princeton,New Jersey,8540	Kelly FitzGerald, PhD, CIP	22-Jul-23
United States	7858	WCG IRB 212 Carnegie Center,Suite 301,Princeton,New Jersey,8540	Kelly FitzGerald, PhD, CIP	23-May-23
United States	7859	WCG IRB 212 Carnegie Center,Suite 301,Princeton,New Jersey,8540	Kelly FitzGerald, PhD, CIP	24-May-23
United States	7860	WCG IRB 212 Carnegie Center,Suite 301,Princeton,New Jersey,8540	Kelly FitzGerald, PhD, CIP	24-Apr-23
United States	7862	WCG IRB 212 Carnegie Center,Suite 301,Princeton,New Jersey,8540	Kelly FitzGerald, PhD, CIP	24-Apr-23
United States	7864	University of Buffalo Institutional Review Board 875 Ellicott St,Buffalo,New York,14203	Bonner, Matthew, PhD Cimino, Michael MS, RPh, Ryan, A. John, MD, ,	27-Jul-23
United States	7865	WCG IRB 212 Carnegie Center,Suite 301,Princeton,New Jersey,8540	Kelly FitzGerald, PhD, CIP	16-Jun-23
United States	7866	WCG IRB 212 Carnegie Center,Suite 301,Princeton,New Jersey,8540	Kelly FitzGerald, PhD, CIP	14-Jun-23

Country	Site no.	Name and address of IEC/IRB	Chairman of IEC/IRB	Date of Approval
United States	7867	WCG IRB 212 Carnegie Center,Suite 301,Princeton,New Jersey,8540	Kelly FitzGerald, PhD, CIP	17-May-23
United States	7868	WCG IRB 212 Carnegie Center,Suite 301,Princeton,New Jersey,8540	Kelly FitzGerald, PhD, CIP	02-Jun-23
United States	7869	WCG IRB 212 Carnegie Center,Suite 301,Princeton,New Jersey,8540	Kelly FitzGerald, PhD, CIP	24-May-23
United States	7870	WCG IRB 212 Carnegie Center,Suite 301,Princeton,New Jersey,8540	Kelly FitzGerald, PhD, CIP	16-May-23
United States	7872	WCG IRB 212 Carnegie Center,Suite 301,Princeton,New Jersey,8540	Kelly FitzGerald, PhD, CIP	26-May-23
United States	7873	WCG IRB 212 Carnegie Center,Suite 301,Princeton,New Jersey,8540	Kelly FitzGerald, PhD, CIP	18-May-23
United States	7875	WCG IRB 212 Carnegie Center,Suite 301,Princeton,New Jersey,8540	Kelly FitzGerald, PhD, CIP	16-Aug-23
United States	7876	WCG IRB 212 Carnegie Center,Suite 301,Princeton,New Jersey,8540	Kelly FitzGerald, PhD, CIP	15-May-23

Country	Site no.	Name and address of IEC/IRB	Chairman of IEC/IRB	Date of Approval
United States	7877	WCG IRB 212 Carnegie Center,Suite 301,Princeton,New Jersey,8540	Kelly FitzGerald, PhD, CIP	17-Jul-23
United States	7878	WCG IRB 212 Carnegie Center,Suite 301,Princeton,New Jersey,8540	Kelly FitzGerald, PhD, CIP	15-May-23
United States	7879	WCG IRB 212 Carnegie Center,Suite 301,Princeton,New Jersey,8540	Kelly FitzGerald, PhD, CIP	24-May-23
United States	7880	WCG IRB 212 Carnegie Center,Suite 301,Princeton,New Jersey,8540	Kelly FitzGerald, PhD, CIP	15-Jun-23
United States	7882	WCG IRB 212 Carnegie Center,Suite 301,Princeton,New Jersey,8540	Kelly FitzGerald, PhD, CIP	23-May-23
United States	7883	WCG IRB 212 Carnegie Center,Suite 301,Princeton,New Jersey,8540	Kelly FitzGerald, PhD, CIP	29-Aug-23
United States	7884	WCG IRB 212 Carnegie Center,Suite 301,Princeton,New Jersey,8540	Kelly FitzGerald, PhD, CIP	26-May-23
United States	7885	WCG IRB 212 Carnegie Center,Suite 301,Princeton,New Jersey,8540	Kelly FitzGerald, PhD, CIP	21-Jul-23

Country	Site no.	Name and address of IEC/IRB	Chairman of IEC/IRB	Date of Approval
United States	7886	Lehigh Valley Health Network Institutional Review Board 6T-31 17th & Chew Streets,Research Participant Protection Office,Allentown,Pennsylvania,18102	Leroy Kromis, PharmD	27-Jul-23
United States	7887	WCG IRB 212 Carnegie Center,Suite 301,Princeton,New Jersey,8540	Kelly FitzGerald, PhD, CIP	07-Jul-23
United States	7888	WCG IRB 212 Carnegie Center,Suite 301,Princeton,New Jersey,8540	Kelly FitzGerald, PhD, CIP	20-Sep-23
United States	7890	WCG IRB 212 Carnegie Center,Suite 301,Princeton,New Jersey,8540	Kelly FitzGerald, PhD, CIP	07-Jun-23
United States	7892	WCG IRB 212 Carnegie Center,Suite 301,Princeton,New Jersey,8540	Kelly FitzGerald, PhD, CIP	26-Jun-23
United States	7893	WCG IRB 212 Carnegie Center,Suite 301,Princeton,New Jersey,8540	Kelly FitzGerald, PhD, CIP	23-Jun-23
United States	7897	WCG IRB 212 Carnegie Center,Suite 301,Princeton,New Jersey,8540	Kelly FitzGerald, PhD, CIP	21-Sep-23
United States	7899	WCG IRB 212 Carnegie Center,Suite 301,Princeton,New Jersey,8540	Kelly FitzGerald, PhD, CIP	30-Jun-23

Country	Site no.	Name and address of IEC/IRB	Chairman of IEC/IRB	Date of Approval
United States	7900	WCG IRB 212 Carnegie Center,Suite 301,Princeton,New Jersey,8540	Kelly FitzGerald, PhD, CIP	10-Oct-23
United States	7901	WCG IRB 212 Carnegie Center,Suite 301,Princeton,New Jersey,8540	Kelly FitzGerald, PhD, CIP	06-Jul-23
United States	7902	WCG IRB 212 Carnegie Center,Suite 301,Princeton,New Jersey,8540	Kelly FitzGerald, PhD, CIP	15-Feb-2023
United States	7907	WCG IRB 212 Carnegie Center,Suite 301,Princeton,New Jersey,8540	Kelly FitzGerald, PhD, CIP	15-Feb-2023
United States	7908	CEP-ISD - Instituto de Ensino e Pesquisa Alberto Santos Dumont Av. Santos Dumont,n 1560,2º andar, sala 24,Macaíba,Rio Grande do Norte,59280-000	Kelly FitzGerald, PhD, CIP	17-Jun-23
United States	7909	WCG IRB 212 Carnegie Center,Suite 301,Princeton,New Jersey,8540	Kelly FitzGerald, PhD, CIP	14-Jul-23
United States	7910	WCG IRB 212 Carnegie Center,Suite 301,Princeton,New Jersey,8540	Kelly FitzGerald, PhD, CIP	28-Jun-23
United States	7911	WCG IRB 212 Carnegie Center,Suite 301,Princeton,New Jersey,8540	Kelly FitzGerald, PhD, CIP	30-Jun-23

Country	Site no.	Name and address of IEC/IRB	Chairman of IEC/IRB	Date of Approval
United States	7912	WCG IRB 212 Carnegie Center,Suite 301,Princeton,New Jersey,8540	Kelly FitzGerald, PhD, CIP	28-Jun-23
United States	7914	WCG IRB 212 Carnegie Center,Suite 301,Princeton,New Jersey,8540	Kelly FitzGerald, PhD, CIP	01-Sep-23
United States	7916	WCG IRB 212 Carnegie Center,Suite 301,Princeton,New Jersey,8540	Kelly FitzGerald, PhD, CIP	07-Sep-23
United States	7918	WCG IRB 212 Carnegie Center,Suite 301,Princeton,New Jersey,8540	Kelly FitzGerald, PhD, CIP	18-Jul-23
United States	7919	WCG IRB 212 Carnegie Center,Suite 301,Princeton,New Jersey,8540	Kelly FitzGerald, PhD, CIP	28-Jul-23
United States	7920	WCG IRB 212 Carnegie Center,Suite 301,Princeton,New Jersey,8540	Kelly FitzGerald, PhD, CIP	15-Aug-23
United States	7921	WCG IRB 212 Carnegie Center,Suite 301,Princeton,New Jersey,8540	Kelly FitzGerald, PhD, CIP	22-Jun-23
United States	7922	WCG IRB 212 Carnegie Center,Suite 301,Princeton,New Jersey,8540	Kelly FitzGerald, PhD, CIP	21-Jun-23

Country	Site no.	Name and address of IEC/IRB	Chairman of IEC/IRB	Date of Approval
United States	7929	WCG IRB 212 Carnegie Center,Suite 301,Princeton,New Jersey,8540	Kelly FitzGerald, PhD, CIP	17-Jul-23
United States	7930	WCG IRB 212 Carnegie Center,Suite 301,Princeton,New Jersey,8540	Kelly FitzGerald, PhD, CIP	07-Jul-23
United States	7931	WCG IRB 212 Carnegie Center,Suite 301,Princeton,New Jersey,8540	Kelly FitzGerald, PhD, CIP	31-Aug-23
United States	7934	WCG IRB 212 Carnegie Center,Suite 301,Princeton,New Jersey,8540	Kelly FitzGerald, PhD, CIP	26-Jul-23
United States	7935	WCG IRB 212 Carnegie Center,Suite 301,Princeton,New Jersey,8540	Kelly FitzGerald, PhD, CIP	06-Jul-23
United States	7936	WCG IRB 212 Carnegie Center,Suite 301,Princeton,New Jersey,8540	Kelly FitzGerald, PhD, CIP	16-Aug-23
United States	7939	WCG IRB 212 Carnegie Center,Suite 301,Princeton,New Jersey,8540	Kelly FitzGerald, PhD, CIP	21-Aug-23
United States	7940	WCG IRB 212 Carnegie Center,Suite 301,Princeton,New Jersey,8540	Kelly FitzGerald, PhD, CIP	05-Aug-23

Country	Site no.	Name and address of IEC/IRB	Chairman of IEC/IRB	Date of Approval
United States	7943	WCG IRB 212 Carnegie Center,Suite 301,Princeton,New Jersey,8540	Kelly FitzGerald, PhD, CIP	10-Jul-23
United States	7949	WCG IRB 212 Carnegie Center,Suite 301,Princeton,New Jersey,8540	Kelly FitzGerald, PhD, CIP	31-Aug-23
United States	7950	WCG IRB 212 Carnegie Center,Suite 301,Princeton,New Jersey,8540	Kelly FitzGerald, PhD, CIP	09-Aug-23
United States	7959	WCG IRB 212 Carnegie Center,Suite 301,Princeton,New Jersey,8540	Kelly FitzGerald, PhD, CIP	13-Jul-23
Vietnam	8402	Tam Anh TP Ho Chi Minh General Hospital 2B Pho Quang street, ward 2,Tan Binh district,Ho Chi Minh city,700000	Assoc.Prof. Pham Nguyen Vinh, PhD., Md.	07-Apr-23
Vietnam	8402	Ethics Committee in Biomedical Research under Tam Anh TP Ho Chi Minh General Hospital 2B Pho Quang Street, Ward 2, Tan Binh District.,Ho Chi Minh,0	Assoc.Prof. Pham Nguyen Vinh, PhD., Md.	18-Sep-23
Vietnam	8410	Ethics Committee in Biomedical Research under Tam Anh General Hospital 108 Hoang Nhu Tiep Street, Bo De ward, Long Bien District,Hanoi,100000	GS. TS. BS. Ngo Quy Chau	25-Apr-23

Country	Site no.	Name and address of IEC/IRB	Chairman of IEC/IRB	Date of Approval
United Arab Emirates	8901	SKMC Institutional Review Board/ Ethics Committee<br>Al Karamah Street, Al Wahda, Abu Dhabi, UAE,Abu Dhabi,51900	Kamran Ahmed	17-Jul-23
United Arab Emirates	8902	Sheikh Khalifa Medical City Institutional Review Board<br>Karama Street,Sheikh Khalifa Medical City,Abu Dhabi,51900	Kamran Ahmed	17-Jul-23

16.1.3.2 List of Global Informed Consent Forms

Global ICF Name and Version No	Version Date
Master ICF Phase III - V1.0	13-Oct-22
Master ICF Phase III - V2.0	02-Dec-22
Master ICF Phase III - V3.0	14-Feb-23
Master ICF Phase III - V4.0	30-Jun-23
Parental ICF Phase III - V1.0	13-Oct-22
Parental ICF Phase III - V2.0	02-Dec-22
Parental ICF Phase III - V3.0	14-Feb-23
Parental ICF Phase III - V4.0	30-Jun-23
Assent ICF (12-17 years) - V1.0	13-Oct-22
Assent ICF (12-17 years) - V2.0	02-Dec-22
Assent ICF (12-17 years) - V3.0	14-Feb-23
Assent ICF (12-17 years) - V4.0	26-Jun-23
